# The Role of Electrostatic Interactions in IFIT5-RNA Complexes Predicted by the UBDB+EPMM Method

**DOI:** 10.1021/acs.jpcb.2c04519

**Published:** 2022-11-03

**Authors:** Urszula Anna Budniak, Natalia Katarzyna Karolak, Marta Kulik, Krzysztof Młynarczyk, Maria Wiktoria Górna, Paulina Maria Dominiak

**Affiliations:** † Biological and Chemical Research Centre, Department of Chemistry, University of Warsaw, ul. Żwirki i Wigury 101, 02-089 Warszawa, Poland; ‡ Nencki Institute of Experimental Biology, Polish Academy of Sciences, ul. Ludwika Pasteura 3, 02-093 Warszawa, Poland

## Abstract

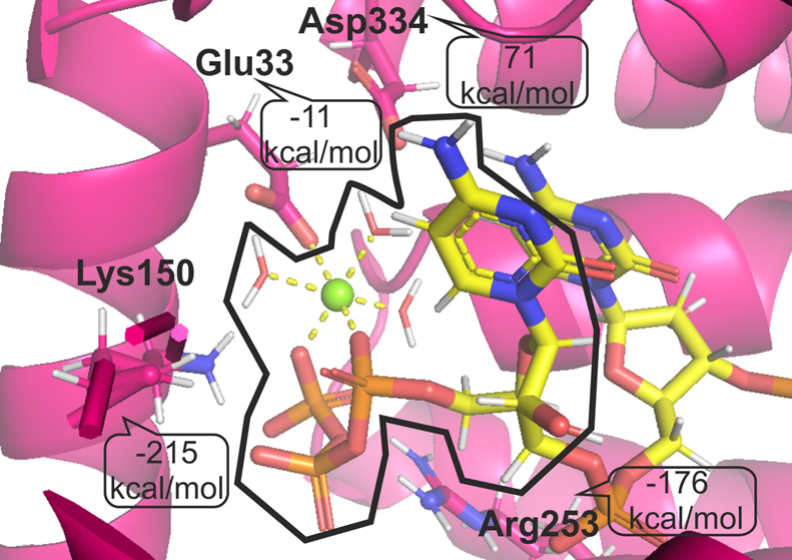

Electrostatic energy has a significant contribution to intermolecular interaction energy, especially in biological systems. Unfortunately, precise quantum mechanics calculations are not feasible for large biological systems; hence, simpler calculation methods are required. We propose a method called UBDB+EPMM (University at Buffalo Pseudoatom DataBank + Exact Potential Multipole Moments), which shortens computational time without losing accuracy. Here, we characterize electrostatic interactions in selected complexes of IFIT proteins with RNA. IFIT proteins are effectors of the innate immune system, and by binding foreign RNA, they prevent the synthesis of viral proteins in human host cells; hence, they block the propagation of viruses. We show that by using the UBDB+EPMM method it is possible to describe protein-RNA interactions not only qualitatively but also quantitatively. Looking at the charge penetration contribution to electrostatic interactions, we find all amino acid residues with strong local interactions. Moreover, we confirm that electrostatic interaction of IFIT5 with pppRNA does not depend on the sequence of the RNA.

## 1 Introduction

RNA binding and recognition are essential for proteins working on RNA in both transcription and translation as well as RNA degradation. The binding events between proteins and RNA rely largely on electrostatics when the molecules approach each other, since these require long-range interactions. It is expected that steric requirements and weaker, more directional interactions such as hydrogen bonds add to the binding specificity at a closer range when the forming protein-RNA complex is being fine-tuned. Apart from guiding the spatial orientation of interacting molecules, electrostatics remains a major contribution to the binding affinity in general.^1^ One of the main sources of charge in these interactions is phosphates of the RNA backbone. The interfaces of proteins interacting with RNA are enriched for polar and charged amino acids, especially the positively charged Arg and Lys, which is in line with the common role of the negatively charged phosphate backbone in these interactions.^2^ In addition, the natural 5′ end of RNA bears a triphosphate moiety (pppRNA) which can be modified by capping with m^7^G in the case of eukaryotic mRNA. Some viruses leave unmodified ends on their genomic RNA or mRNA; for example, the ssRNA(−) genomes of *Vesicular Stomatitis* or *Influenza* viruses bear triphosphate groups.^3^ Due to RNA capping or processing occurring in the nucleus, unmodified pppRNA should normally be absent from the cytosol of human cells and thus can be recognized by cytosolic receptors as pathogen-associated molecular patterns (PAMPs) and trigger antiviral response.^4^

pppRNA recognition is crucial in antiviral defense: dsRNA is recognized by the RIG-I receptor which initiates the antiviral signaling cascade, while ssRNA is recognized by the effector protein IFIT5. IFIT5 is unique since it lacks enzymatic activity and reportedly acts by sequestering pppRNA to prevent viral replication or translation. IFIT5 binding to pppRNA is demonstrated in detail by three cocrystal structures.^5^ In line with the ability of IFIT5 to distinguish pppRNA from other types of RNA (for example, monophosphorylated pRNA which is a common RNA degradation product), the ppp group is especially important for binding affinity and thus conveys selectivity toward pppRNA. Crucial for this interaction is a Mg^2+^ ion, which is a common metal used by RNA binding proteins and a key counterion relevant for RNA folding and interactions.^6^ The requirement for an unpaired 5′ end comes from the restrictive dimensions of the RNA binding site in IFIT5 which allow only ssRNA with at least a 4–5 nucleotide unpaired 5′ overhang. IFIT5 was able to cocrystallize with three different artificial homooligonucleotides (oligoU, oligoA, and oligoC). In these crystal structures, the nucleobases form very few hydrogen bonds with IFIT5, based on which it was postulated that IFIT5 can bind RNA of any sequence. The role of RNA sequence in binding to IFIT5 has been experimentally addressed to a very limited extent by RNA binding studies.^7^ The studies typically focused more on varying 5′ moieties rather than RNA sequence. Literature reports are not consistent about the selectivity of IFIT proteins and their preferred RNA forms.^5,8−10^ Thus, the common assumption, that IFIT5 binds RNA regardless of the nucleotide sequence, is mostly based on the existence of the aforementioned crystallographic structures in PDB and merits further quantitative examination. Characterization of interactions between IFIT5 protein and RNA would help us to understand the mechanism and selectivity of recognition of viral RNA. If IFIT5 proteins indeed do not discriminate against RNA sequence, it would indicate that the mechanism of virus detection is universal (sequence-agnostic) in immune systems, which in turn would suggest that viral 5′ ends might occur with any sequence. On the contrary, if IFIT5 displays any preferences for particular sequences of pppRNA, it could mean that they take part in the recognition of specific kinds of viral RNA or that the 5′ end sequence repertoire of viruses is limited. In this study, we make use of the available high-quality crystal structures of IFIT5 with different RNA to address the question of rules regarding the specificity of IFIT5 and more generally of RNA binding proteins.

Molecular modeling plays a crucial role in drug design, understanding mechanisms of biological processes, and specifying structure–function relations in proteins. Determination of structure and interaction energy is essential in the prediction of new drugs or the analysis of protein complexes. Biomacromolecules (proteins, nucleic acids) are too complex systems for precise quantum mechanical computations. At the same time, information about the energy of their interactions is desired. Electrostatic energy has usually the most significant contribution to interaction energy (especially in the biological systems) and is a key factor in processes of molecular recognition, protein stabilization, or protein folding. It has been shown that electrostatic energy is a sufficient approximation of total interaction energy.^11^ Furthermore, it is the most robust component of total energy, because it is the least sensitive to errors in the geometry of investigated systems. Such errors are often encountered while determining structures of big, biological complexes, for which it is very difficult to properly define the positions of all atoms. There are affordable computational methods, which enable estimation of the energy of electrostatic interactions in macromolecules; however, most of them rely on simplified methods taken from classical mechanics (force fields). In classical molecular mechanics, electrostatic interactions are usually approximated by simple Coulomb interactions of atomic point charges. To estimate electrostatic energies of interactions in IFIT-RNA complexes, we used a more sophisticated method called the University at Buffalo Pseudoatom DataBank (UBDB)^12,13^ plus the Exact Potential Multipole Moments (EPMM) method.^14^

In the UBDB+EPMM method, the continuous aspherical model of charge density is used. Using continuous charge density instead of point charges used in many force fields, charge penetration effects are taken into account. With the UBDB+EPMM method, it is possible to compute electrostatic energies with similar accuracy as with quantum chemistry methods, for a wide range of types of interactions (hydrogen bonds, π–π stacking) and distances (not only at equilibrium geometry but also below or above).^15^ The UBDB+EPMM method was verified on many occasions for various compounds (small organic molecules, amino acids, nucleobases) and compared with quantum chemical results and molecular mechanics. It reproduces well the electrostatic energies obtained from quantum chemical calculations, with the RMS difference not larger than 5 kcal/mol, depending mainly on the type of interacting molecules and the reference method.^12,13,16,17^ The UBDB+EPMM approach was also tested on larger benchmark sets (S66^18^ and JSCH-2005^19^) and compared to the force field energies.^15,20^ The S66 set represents the most common noncovalent interactions observed in biological structures. Results obtained for S66 showed that the RMSE between the UBDB+EPMM method and the reference B3LYP/aug-cc-pVTZ method equaled only 1.1 kcal/mol.

The UBDB is a databank of aspherical atomic electron densities derived by Fourier-space fitting of the Hansen-Coppens Multipole Model (HCMM)^21^ of pseudoatoms to molecular electron densities obtained from DFT calculations.^12^ The calculations are done for a couple of thousands of model molecules, and the resulting tens of thousands of pseudoatoms are grouped into atoms having similar values of electron density parameters and similar chemical topologies. Based on each group, an atom type is defined, and characteristics for those atom type parameters of atomic electron density are stored in the databank, in the form of the HCMM parameters. The UBDB was recently restructured, extended, and renamed the Multipolar Atom Types from Theory and Statistical clustering (MATTS) databank.^13^ The idea of pseudoatom databanks comes from X-ray crystallography, where the databanks become to be widely used for crystal structure refinements replacing the Independent Atom Model (IAM). IAM is a standard model of electron density commonly used in crystallography. In IAM, individual atoms are represented by the spherically averaged electron densities obtained by quantum mechanics methods for isolated atoms in the ground state. To obtain more accurate electron densities, more sophisticated models were designed by quantum crystallography. The HCMM is one such model. In HCMM, atoms are represented with a finite spherical harmonic expansion (called pseudoatoms) of the electron density around each atomic center. The electron density of a pseudoatom is defined by eq 1.Hansen-Coppens formalism used for charge density analysis
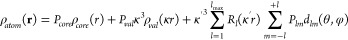
1In this equation, ρ_*core*_ and ρ_*val*_ are free-atom core and valence spherical densities normalized to one electron, *R*_*l*_ is the Slater-type radial function, and *d*_*lm*_ are density-normalized real spherical harmonic functions. The third term is responsible for aspherical deformations. κ and κ′ are expansion-contraction parameters, and *P*_*core*_, *P*_*val*_, and *P*_*lm*_ are core, valence, and spherical harmonics populations, respectively.

With UBDB, it is possible to reconstruct electron density from parameters *P*_*val*_, *P*_*lm*_, κ, and κ′ transferred from the databank to the studied molecule for which the only input information is coordinates of atoms. The reconstructed UBDB model provides quantitative information about the electron density and enables the performing of topological analysis of electron density on the basis of Bader’s QTAIM theory (Quantum Theory of Atoms In Molecules).^22^ In the UBDB approach, one can also compute various atomic and molecular properties such as charge, dipole and higher moments, molecular electrostatic potential, and electrostatic energy of interactions between molecules.

The EPMM method applied during the computation of electrostatic energies from the UBDB electron densities shortens computational time by combining two different approaches. The EPMM method evaluates the exact Coulomb integral in the inner region (≤4.5 Å) (EP) and combines it with a Buckingham-type multipole moments approximation (MM) for long-range interatomic interactions.^14^ Using the EPMM method, it is possible to take into account the penetration contribution to the electrostatic energy. The penetration effect occurs when two molecules are so close that their electron densities are overlapping and the computation of electrostatic interaction energy from multipole moments is no longer valid. Penetration energy is defined as a difference between the exact electrostatic interaction energy computed by integration over the continuous distributions of charge (here EPMM) and the electrostatic interaction energy computed from multipole moments (here MM).

The UBDB+EPMM method has been successfully used in previous research to analyze interactions in peptides, proteins, and various complexes, e.g., aminoglycosides with RNA.^23−26^ Here, we will investigate for the first time complexes of proteins with RNA. Moreover, the studied complexes contain magnesium ions, which mediate the protein-RNA interactions. Our study provides a deeper understanding of electrostatics aspects of protein–RNA interactions and additional insight into the specificity of the antiviral IFIT5 protein toward various features of RNA.

## 2 Materials and Methods

### 2.1 Electrostatic Properties Calculations

Calculations for IFIT5-RNA complexes were based on the following structures deposited in the RCSB Protein Data Bank:^5^4HOR, 4HOS, and 4HOT. For proper calculations, deposited structures were thoroughly checked, and some adjustments were done. The 4HOS structure originally contains a sodium ion in the binding site, instead of a magnesium ion. The geometry of the binding site of the 4HOS structure allows us to place there other metal ligands, e.g., calcium, which was confirmed by the Checkmymetal server.^27^ The magnesium ion is not the most preferable cation, but it is not forbidden. For the purpose of this work, we changed the metal in the binding site of the 4HOS structure to magnesium. Hydrogen atoms were not present in any deposited structures of IFIT5-RNA complexes. We used the Molprobity program^28^ to add hydrogens atoms. Hydrogen atoms were positioned according to standard neutron distances. All flips suggested by the program were accepted. In the 4HOR structure, two residues were flipped: Asn346 and Gln462; in the 4HOS structure, four residues were flipped: Gln40, Gln76, Asn346, and His398; and in the 4HOT structure, two residues were flipped: His85 and Gln377. Both in 4HOR and 4HOT structures all histidine residues were neutral, in contrast to 4HOS in which His62 and His399 were protonated. After adding hydrogens, water molecules have been removed except for the three in the coordination sphere of magnesium. Hydrogen atoms for the three water molecules were added geometrically (Table S3) on the basis of analogous small molecule structures containing magnesium coordinated by water. One was (bis(ethane-1,2-diammonium) diaqua-bis(hydrogen diphosphato)-magnesium deposited in the Cambridge Structural Database^29^ under the refcode GEQBIO.^30^ The second was magnesium bis(dihydrogen phosphate(I)) hexahydrate deposited in the Inorganic Crystal Structure Database^31^ under the code 2549.^32^ The whole IFIT5 protein consists of 482 amino acid residues. In all the deposited structures, some residues at the N- or the C-termini of the proteins were missing. The largest number of missing amino acid residues was in the complex with the oligoA (4HOT): Met1, Ser2, Glu3, Ile4, Arg5, and Ile482. Also in the complex with the oligoU (4HOS), the RNA chain is shorter than in the other two complexes; only the first three nucleotides are fully described, and the fourth one is incomplete. Therefore, for the proper calculations and to be able to compare all three complexes, we selected residues 7–480 from the protein chain and the first three residues from the RNA chain for further analyses. We performed analyses for both, the whole protein and the protein binding site only. The binding site was chosen on the basis of the geometrical analysis of all three complexes with previously added hydrogen atoms. We selected all amino acid residues within a 5 Å distance from the first three RNA residues including the magnesium ion and water molecules coordinating it. Selected amino acid residues are listed in the SI (Supplementary File SI2). The nucleobase in the second nucleotide (C2) in the RNA from the 4HOR structure is disordered over two conformers, the *syn* and *anti* conformation of the nucleobase. Both conformers were taken independently into account, but for expedience, we present the detailed electrostatic analysis focusing on the main conformer, which is the *syn* configuration. The *anti* conformer was only used for energy comparison with results from molecular dynamics simulations.

The LSDB program^33^ was used to transfer electron density parameters from the UBDB databank to investigated structures. For the proper transfer, the LSDB program was used twice for each structure. First, the program was used only to extend the position of hydrogen atoms to neutron distances (with the RADII command for Mg set to 0.01 Å to remove Mg–O bonds from the procedure). Second, the databank was transferred; during the transfer, the RADII command for Mg was set at the default value (1.36 Å), and H atoms were not shifted. The extended version of UBDB from the 2012 year was used.^12^ The extension regarded new atom types needed to describe the magnesium cation and its neighbors. Calculations for new atoms types were based on the structures deposited in two databases: Cambridge Structural Database^29^ and Inorganic Crystal Structure Database.^31^ For the calculations, homemade scripts routinely used for building the UBDB databank were adapted and used. In particular, the multipolar refinement was modified, and the κ′ parameters for Mg were not refined. More details about computing and verifying magnesium-related atom types are given in the SI. After the UBDB transfer, each residue was individually scaled to its formal charge. The exception was made for the first residue of each RNA structure (CTP1, UTP1, and ATP1), Glu33 from the protein chain, and the magnesium cation with three water molecules coordinating it. They were scaled together as one fragment to a charge of −3 e. Electrostatic interaction energies were calculated for each possible pair of an amino acid residue and one of the three first RNA residues using homemade scripts to prepare input files and analyze output files. The magnesium cation and the three water molecules were considered as a part of the first RNA fragment for electrostatic energy calculations. Two types of methods were used to compute energies, EPMM and aMM,^14^ both implemented in the XDPROP module of the XD2016 package.^34^ From the EPMM-aMM difference, the penetration contribution to electrostatic interaction energy (Epen) was calculated. Spherical core and valence electron densities were computed from the atomic Clementi and Roetti^35^ wave functions. Single-zeta Slater functions for the deformation part were taken from Clementi and Raimondi^36^ with default values of n(l) except for P and S atoms, for which the n(l = 1,2,3,4) equal to 2,4,6,8^37^ and 6,6,6,6^38^ were applied, respectively. Exact integration parameters were set to iqt = 2, Nrad = 99, and Nang = 590, and the exact potential zone radius (rcrit1) was set to 5 Å.

The average time of transferring the UBDB for one protein-RNA complex is around 4 min. The average time of computing electrostatic interaction energies for one protein with one nucleotide is between 13 to 40 min for the EPMM method depending on the number of interactions integrated (interatomic distances closer than 5 Å) and 15 s for the MM method. Calculations were performed on a PC with an Intel(R) Core(TM) i7-7700 CPU @ 3.60 GHz processor, 32 GB RAM, and Windows10 Pro system.

Calculated electrostatic interaction energies for all amino acid residues are deposited in the supplementary file “SI_Energies”.

Electrostatic potential maps were calculated from the UBDB-derived electron densities in the XDPROP module of the XD2016 package and visualized in the MoleCoolQt64 program.^39^ For RNA maps, cubic grids were generated with the size of a box 500 × 500 × 500 Å and voxel size 0.1 Å centered on the magnesium cation. For the protein maps, the procedure was similar to the difference in the size of the box equal to 250 × 250 × 250 Å and the voxel size 0.5 Å. Figures were prepared in PyMol.^40^

### 2.2 Molecular Dynamics Simulations

Five systems containing IFIT5 proteins were chosen for simulations. Two systems with IFIT5-RNA complexes were based on the 4HOR structure^5^ with one additional cytosine residue at the 3′-end of RNA. We considered two conformational states of the second nucleobase with the torsion angles χ (O4′–C1′–N1–C2) equal to −104° and 77°, corresponding to the conformations *anti* and *syn*, named IFIT5-ppp5C(C2*anti*) and IFIT5-ppp5C(C2*syn*), respectively (Figure S2). The next system consisted of the 4HOT structure,^5^ extended with 8 adenines at the 3′-end of the RNA, named further as the IFIT5-ppp12A system. Additionally, two systems without ligands were based on the 4HOQ structure^5^ with and without the protonation of Asp334. The Mg^2+^ ions and all the water molecules present in the crystal structures were preserved. The YASARA v.20.4 program^41^ was used for modeling the missing protein fragments and adding the hydrogen atoms at pH 7.4, taking into account the hydrogen bonds optimization. The amino acid protonation states were assessed with PROPKA v 3.0.4.^42^ All systems were solvated in a dodecahedral box of water molecules. The salt concentration was set to a physiological level of 150 mM NaCl. Each of the modeled systems contained about 93,000 atoms, except for the IFIT5-ppp12A system, which contained more than 180,000 atoms. The CHARMM36m force field^43^ and the TIP3P water model^44^ were used. Simulations and analyses were performed with GROMACS 2016.5.^45^ A cutoff of 12 Å and a 10–12 Å F-switch were applied for the van der Waals and short-range electrostatic interactions. The Particle Mesh Ewald method^46^ was used to handle the long-range electrostatic interactions.

The LINCS algorithm was used to constrain the bonds involving hydrogen atoms.^47^ The velocity-rescale thermostat^48^ and Parrinello–Rahman barostat^49^ were used to regulate the temperature and pressure. Short energy minimization was done with the steepest descent algorithm. The whole system was equilibrated in the NVT ensemble for 50 ps with weak positional restraints applied on all heavy atoms and in the NPT ensemble in three stages for 50, 25, and 25 ps, gradually increasing the temperature to the final value of 310.15 K. The production simulation, with 2 fs time step, was conducted for 1 μs. For each system, 3 independent simulation runs were performed, which gives the total production simulation time equal to 15 μs. The analysis of the trajectories was done using GROMACS 2016.5 tools and Python 3.6 scripts. Figures were prepared in Chimera.^50^

Two systems, IFIT5-ppp5C(C2*anti*) and IFIT5-ppp5C(C2*syn*), were chosen for electrostatic energy calculations using the UBDB+EPMM method and the calculations using the Coulomb equation with the point charges taken from the CHARMM36m force field and the TIP3P water model. For each simulation run, we chose a single representative of the biggest cluster (single frame selected from the simulation run that is the closest to the average structure). Then, the electrostatic energy calculations were conducted with the UBDB+EPMM or MM methods, as it was described in section 2.1, or using in-house scripts in the case of the point charges. Calculated electrostatic interaction energies for six simulation runs are deposited in the supplementary file “SI_Energies”.

### 2.3 Biological Experiments

N-term HisTag IFIT5 WT, IFIT5 K150M, and IFIT5 Q41E/K150M/R253M proteins were expressed from pETG10a^51^ vectors in BL21-CodonPlus(DE3)-RIL cells. The expression was performed overnight at 25 °C after adding a 0.5 mM isopropyl-d-1-thiogalactopyranoside solution (IPTG) at OD 0.5–0.6. Bacteria were collected by centrifugation at 4,000 × g, at 4 °C for 20 min. In the case of IFIT5 WT and IFIT5 K150M, cell pellets were resuspended in 50 mM Tris pH 7.5, 1 M NaCl, 20 mM imidazole, 10% glycerol, 1 mM MgCl_2_, and 0.5 mM (tris(2-carboxyethyl)phosphine) (TCEP) supplemented with EDTA-free SigmaFast Protease Inhibitor Cocktail (Merck), DNase I, and lysozyme. After a 1 h incubation, bacteria were sonicated and centrifuged at 48,880 × g for 30 min. The supernatant was applied on a HisTrap HP column (GE Healthcare) equilibrated in the same buffer (without supplementation). IFIT5 proteins were eluted with a gradient of imidazole from 20 to 1 M. Fractions with purified proteins were combined and diluted 10 times with 50 mM Tris pH 7.5, 10% glycerol, and 1 mM MgCl_2_ to decrease NaCl concentration. Samples were then loaded on a HiTrap Heparin HP column (GE Healthcare), pre-equilibrated with 50 mM Tris pH 7.5, 100 mM NaCl, 10% glycerol, 1 mM MgCl_2_, and 0.5 mM TCEP buffer. The elution was performed with a gradient of NaCl from 100 mM to 1 M. Finally, IFIT5 proteins were purified on a Superdex 200 Increase column in 1x Phosphate-buffered saline (PBS), 5% glycerol, 1 mM MgCl_2_ and 0.5 mM TCEP buffer. The proteins were frozen in liquid nitrogen and kept at −80 °C until use.

Part of the IFIT5 WT and IFIT5 Q41E/K150M/R253M proteins were purified as described above with some modification. The buffer used for cell resuspension had 0.5 M NaCl concentration, and after HisTrap HP column purification, the sample was diluted 5 times. In all purification buffers, MgCl_2_ was omitted.

Cy5 labeled RNA was purchased from Futuresynthesis (Poland) with a 5′-ppp-AAAAAGGAAGGU-Cy5 sequence.

Microscale thermophoresis (MST) experiments were performed on a Monolith NT.115 device (Nanotemper Technologies).^52^ The RNA concentration was kept constant at 10 nM. The unlabeled protein was titrated in 1:1 dilutions with the highest concentration of 5/10 μM IFIT5 WT, 10 μM IFIT5 K150M, and 10/50 μM in the case of IFIT5 Q41E/K150M/R253M. The binding reaction was incubated at 22 °C for 10 min. The measurement was carried out at 22 °C with a 60% IR-laser power and 20/60% LED in the MST buffer (PBS, 5% glycerol, 0.5 mM TCEP, 0.05% Tween20, ±1 mM MgCl_2_). For the wild type and triple mutant, the addition of MgCl_2_ showed no improvement over its lack (IFIT5 WT – apparent K_D_ = 12.71 nM, 13.74 nM, 22.97 nM vs 8.87 nM; IFIT5 Q41E/K150M/R253M – apparent K_D_ = 2.341 μM vs 1.698 μM), so all replicates were used for the final calculation. All measurements of IFIT5 K150M were performed in a buffer with 1 mM MgCl_2_. The recorded fluorescence in 0.5–1.5 s was normalized to the fraction bound and analyzed using GraphPad Prism 9.3.0.

## 3 Results and Discussion

In this study, three structures of complexes of IFIT5 proteins with RNA were investigated: PDB entries 4HOR, 4HOS, and 4HOT. All of them contain as a ligand a short chain of RNA but with different nucleobases. 4HOR contains cytosine, 4HOS contains uracil, and 4HOT contains adenosine homooligonucleotides. For the comparison and calculations, the first three nucleotides of each structure were investigated. They will be denoted as pppCCC, pppUUU, and pppAAA, respectively. In the binding of RNA to IFIT5, a magnesium cation is involved in general, but in the 4HOS structure, a sodium cation is present in the binding site instead of magnesium. For the purpose of this study, we replaced sodium with magnesium, as explained in the Materials and Methods section. In all three structures, the metal cation is coordinated by three water molecules, besides protein and RNA.

### 3.1 Structural Characterization of Investigated Complexes

**Figure 1:**
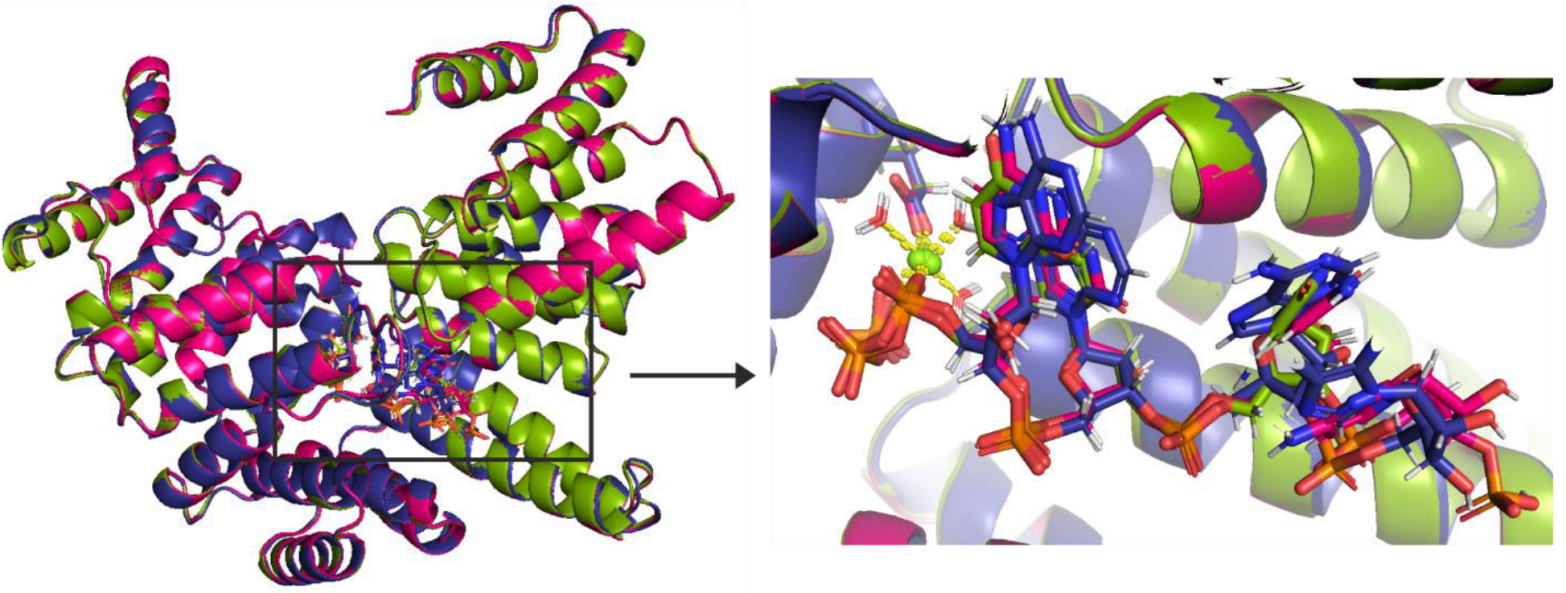
(left) Cα alignment of three investigated structures (4HOR, 4HOS, and 4HOT) taken from the PDB and (right) insight into the binding site. The magenta color denotes the 4HOR structure, the green color denotes the 4HOS structure, and the violet color denotes the 4HOT structure. The green sphere represents the magnesium cation.

All three structures of complexes of IFIT5 with RNA are very similar. The alignment of the protein chains shows no major differences (Figure 1), and the average RMSD for Cα atoms is 0.3 Å. Also, the alignment of RNA chains shows high structural comparability, especially for the first and the second residue. It is worth noting that the nucleobase in the second nucleotide C2, in the pppCCC chain, is found in two conformations, *syn* and *anti* (Figure S2), whereas in the pppUUU chain, the analogous nucleobase is in the *anti* conformation, and in the pppAAA chain, it is in the *syn* conformation.

In this study, we examined the electrostatic energy of the whole protein interacting with the first three residues of the RNA chain. We associated the magnesium cation and water molecules coordinating it with the RNA; thus in fact, we analyzed interactions of IFIT5 with Mg(H_2_O)_3_pppNNN where N is C, U, or A. We paid a special attention to amino acid residues constituting the binding site.

Assignment of charges is crucial in electrostatics calculations. Due to the presence of the triphosphate, the charge of the first residue in the studied RNA chains (pppC being the CTP1 residue, pppU being the UTP1 residue, and pppA being the ATP1 residue) has a formal charge of −4 e, and every next nucleotide has a formal charge of −1 e; but when we take the magnesium cation into consideration, then the Mg(H_2_O)_3_pppN group has the formal charge of only −2 e. The charge of the whole analyzed protein is −2 e for 4HOR and 4HOT and 0 e for 4HOS, but the charge of the binding site is +2 e for all three complexes.

**Figure 2:**
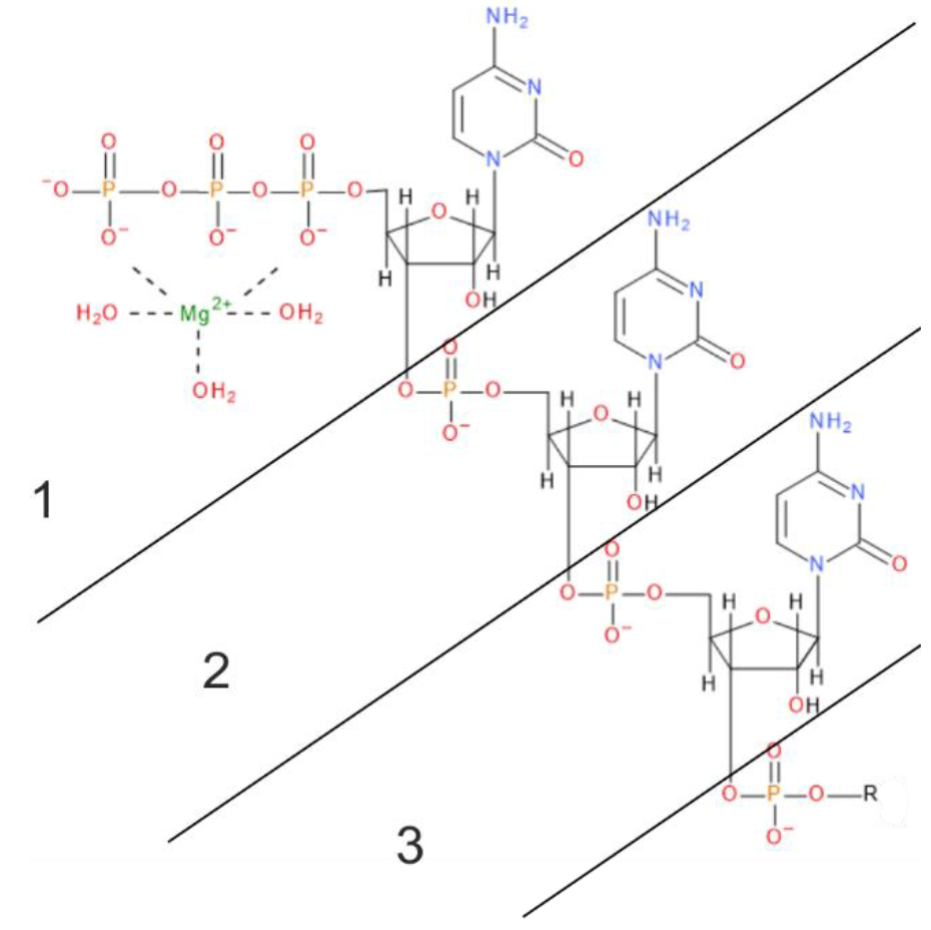
Scheme of the partition of the RNA chain in the 4HOR structure in three fragments. The pppCCC with Mg(H_2_O)_3_^2+^ is shown with formal charges. R states for the rest of the nucleic acid chain.

The magnesium cation is assumed to play a key role in the protein-RNA interactions. In IFIT5-RNA complexes, it has octagonal coordination, being surrounded by the three oxygen atoms from water molecules and three other oxygen atoms. Among the latter, two oxygen atoms are part of the triphosphate group at the 5′-end of the RNA chain (CTP1, UTP1, or ATP1, see Figure 2), and one comes from the protein (from the carboxylic group of the Glu33 side chain). Between the magnesium cation and the Glu33 residue, a coordinate bond is formed, which will be further investigated.

### 3.2 Characterization of Charges and Electrostatic Potential of Investigated Complexes

The magnesium cation is formally charged +2 e in biological complexes. More accurate modeling of charge density often reveals that the formal charge is only a crude approximation.^53^ From our modeling, it appeared that, indeed, the magnesium cation in such complexes as observed in the studied structures has a charge of ca. + 0.26 e. Apparently, some charge transfer from ligands to the magnesium cation is happening when a magnesium coordination complex is formed. In the investigated complexes, the first nucleotide residue with magnesium and three water molecules has the formal charge of −2 e, but the charge calculated from the UBDB model is larger, −2.72 e, see Table 1. The Glu33 residue donates the extra −0.72 e, itself ending up with a charge of −0.28 e (instead of its formal −1 e charge).

**Table 1:** Comparison of Formal and Modeled Charges (e) of the Magnesium Cation and Its Ligands

	formal charge	modeled charge
magnesium cation	+2	+0.26
pppC/pppA/pppU	–4	–3.32
3 water molecules	0	+0.34
Glu33	–1	–0.28
**total**	**–3**	**–3.00**

It is also clearly visible (Table 1) that the triphosphate nucleotides give their electrons (−0.68 e) to the magnesium cation. Even water molecules contribute a little to the lowering of the positive charge of the magnesium cation. Each water molecule in the complex is slightly positive, with a charge of ca. +0.11 e, and all three water molecules are treated as equivalent. The magnesium is not a +2 cation anymore in the IFIT5-RNA complexes but is nearly neutral, and its positive charge is reduced by the ligands.

**Figure 3:**
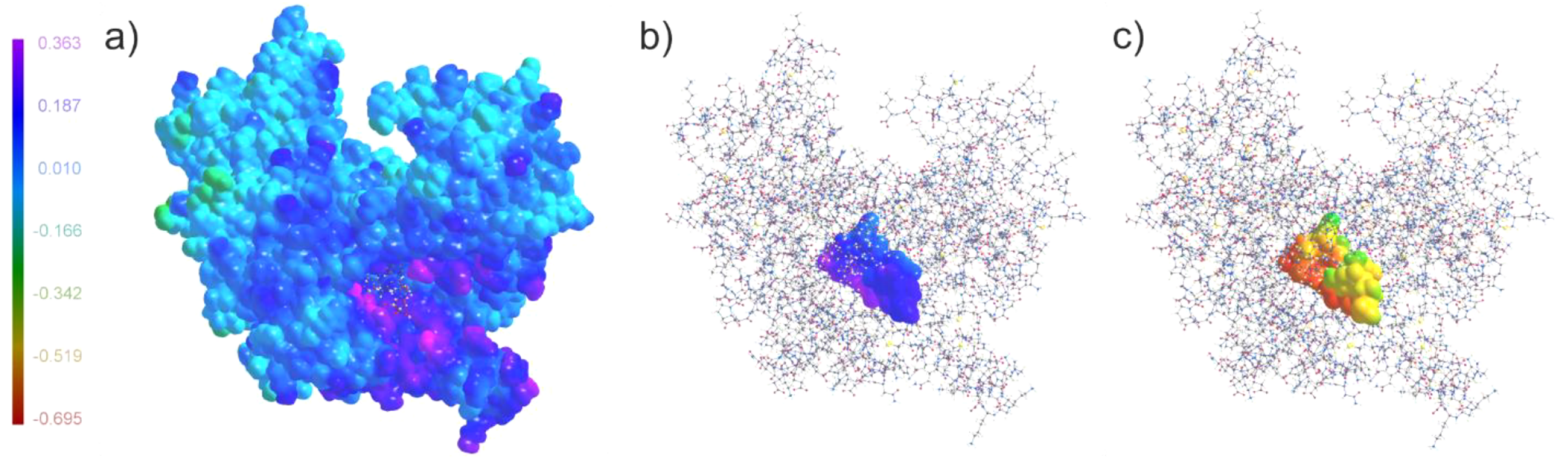
Electrostatic potential (e Bohr^–1^) of the IFIT5 protein and Mg(H_2_O)_3_pppCCCC(C2*syn*) mapped on the isosurface (0.002 e Bohr^–3^) of electron density reconstructed with the UBDB. Color according to the legend. (a) Electrostatic potential of the IFIT5 protein mapped on the isosurface of electron density of IFIT5, (b) electrostatic potential of the IFIT5 protein mapped on the isosurface of electron density of RNA, and (c) electrostatic potential of Mg(H_2_O)_3_pppCCCC(C2*syn*) RNA mapped on the isosurface of electron density of RNA.

The UBDB databank enabled us to calculate electrostatic potential maps from the UBDB-derived electron density instead of deriving them from force field point charges. The electrostatic potential of a protein mapped on its electron iso-density surface shows the concentration of the positive electrostatic potential in the binding site of the protein (Figure 3a). The polarity of the RNA chain is also clearly visible from the maps (Figure 3c). The region of the phosphate backbone of RNA has more negative electrostatic potential than the nucleobases region. The complementarity between the electrostatic potential generated by the protein and electrostatic potential of the RNA molecules is clearly visible from Figures 3b and c.

### 3.3 Electrostatic Interactions

The energies of electrostatic interactions were calculated for every amino acid residue with every RNA fragment by both methods: EPMM and MM. The EPMM is considered a method providing the most accurate electrostatic energy (Ees). From the EPMM-MM difference, the penetration energy (Epen) was calculated. In the case of IFIT5:Mg(H_2_O)_3_pppCCC, the C2 residue in the *syn* conformation was selected for electrostatic interaction analyses.

The charge of investigated RNA chains is always negative. The charge of the whole protein is −2 or 0 e, but it is so distributed that the electrostatic energy of binding RNA with the protein is negative, meaning that overall interaction is attractive. The distribution of the charge of protein and RNA was confirmed by electrostatic potential maps (Figure 3). The total charge for amino acids belonging to the binding site is +2 e for all three complexes; thus, we can observe that the electrostatic energy is higher (more negative) for the binding site than for the whole protein (Table 2). Electrostatic energy is around 1.5 times larger for the binding site than for the whole protein considering the first RNA fragment (Mg(H_2_O)_3_pppN1, where N is C, U, or A), whereas this ratio is around 1.25 for the second nucleotide and around 1 for the third one.

**Table 2:** Electrostatic Interaction Energies [kcal/mol] and Penetration Contributions [kcal/mol] to the Energies Calculated for the Whole Proteins and for the Binding Sites Interacting with Each RNA Fragment ((1-Mg(H_2_O)_3_pppN1, 2-N2, and 3-N3, Where N Stands for C, U, or A)

		Mg(H_2_O)_3_pppCCC				Mg(H_2_O)_3_pppUUU				Mg(H_2_O)_3_pppAAA			
		1	2	3	sum	1	2	3	sum	1	2	3	sum
	charge	–2	–1	–1	–4	–2	–1	–1	–4	–2	–1	–1	–4
		electrostatic interaction energy											
whole protein	–2	–514	–232	–255	–1001					–482	–205	–218	–905
	0					–571	–218	–299	–1087				
binding site	2	–798	–292	–253	–1343	–790	–270	–275	–1335	–767	–274	–232	–1273
		penetration contribution											
whole protein/binding site		–123	–27	–31	–180	–101	–29	–45	–176	–105	–31	–19	–154

Moreover, the first fragment of the RNA has a larger contribution to the binding energy than any next nucleotide. When we analyze interactions with the whole protein, electrostatic energy for the Mg(H_2_O)_3_pppN1 fragment is 2–2.5 times larger than for the second (N2) or the third nucleotide (N3) of the same type. Looking at the binding site only, the ratio is even slightly larger, 2.7–3.2, favoring the first fragment.

Values of Epen listed only once as calculations gave the same values of Epen for the whole protein and the binding site. This fact confirms that the binding site was selected properly, and we included all amino acid residues contributing to the short-distance interactions. For Epen, again a higher contribution for the first RNA fragment is noted, although in this case, it is not the reason for the higher charge of the first fragment but more closer contacts, especially including the contact of the magnesium cation with one of the oxygen atoms from the carboxyl group of Glu33.

Finally, when total Ees energies for Mg(H_2_O)_3_pppCCC, Mg(H_2_O)_3_pppUUU, and Mg(H_2_O)_3_pppAAA are compared to each other, it is clear there is no significant difference between them, the largest discrepancy being about 10%. Thus, energies of electrostatic interactions support the conclusion drawn from the structural analysis of IFIT5 cocrystals with three different homooligomers that IFIT5 proteins bind pppRNAs with similar strength, irrespective of the sequence.^5^ The conclusion regards C, U, and A nucleotides at the first, second, or third position. Nothing is known about interactions with G at these positions because the crystal structure of IFIT5 complexed with guanine pppRNA is unknown.

**Figure 4:**
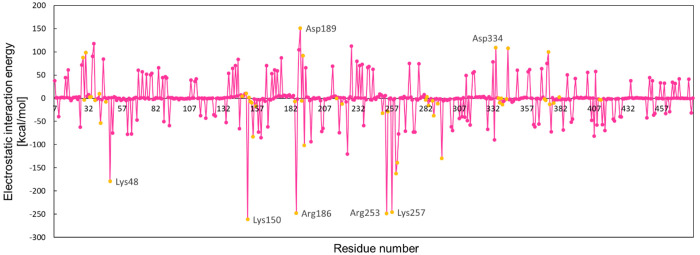
Electrostatic interaction energies Ees [kcal/mol] for particular amino acid residues of the IFIT5 protein interacting with the whole Mg(H_2_O)_3_pppCCC chain (from the 4HOR crystal structure, C2*syn*) marked by the magenta line together with amino acid residues which are situated in the binding site marked by orange.

Figure 4 depicts electrostatic interaction energies between all amino acid residues of the IFIT5 protein with the whole Mg(H_2_O)_3_pppCCC chain. We can observe that, generally, IFIT5 consists of many charged amino acid residues; for the 4HOR structure, it is 68 positively charged and 70 negatively charged amino acids. The amino acid residues interacting with the highest (on absolute value) electrostatic energies are in the binding site, but it is worth mentioning that still many amino acid residues are interacting with high energies outside the binding site.

### 3.4 Detailed Analysis of Interactions

The IFIT5 protein binds RNA via different interactions: long-range electrostatic interactions, hydrogen bonding, short-range van der Waals, stacking, and others. Amino acid residues from protein chains interact with different parts of the RNA chains: nucleobases, sugar moieties, or phosphate bridges. Thanks to our calculations, the detailed analysis of interactions is possible not only on the basis of the geometry but also on the basis of computed energies.

#### 3.4.1 The Complex of IFIT5 with Mg(H_2_O)_3_pppCCC

First, we focused on interactions of amino acids from the binding site of IFIT5 with the first RNA fragment Mg(H_2_O)_3_CTP1, i.e., the first triphosphate nucleotide (CTP1 = pppC) complexed with the magnesium cation and three water molecules. The highest attractive electrostatic interaction energies are observed for interactions of positively charged amino acid residues, Table 3. The highest Ees value is observed for the pair Lys150: Mg(H_2_O)_3_CTP1, and it equals −214.6 kcal/mol. Positively charged Lys150 is situated close to the highly negative triphosphate bridge of the first RNA residue, thus a very strong attractive interaction. For this pair, also penetration energy is high and equals −14.2 kcal/mol. A very strong interaction is the result of four hydrogen bonds (HBs), three between the −NH_3_^+^ group and phosphate and one between −NH_3_^+^ and a water molecule coordinating the magnesium cation, enhanced by charge. The other strongest interactions are as follows: Arg253: Mg(H_2_O)_3_CTP1 (−176.1 kcal/mol), Arg186: Mg(H_2_O)_3_CTP1 (−159.7 kcal/mol), Lys48: Mg(H_2_O)_3_CTP1 (−133.1 kcal/mol). For Arg253, we can also see strong penetration (−10.7 kcal/mol) due to two HBs. Arg186 has a small penetration contribution (−1.5 kcal/mol) due to one HB, and the Lys48 interaction has no penetration contribution at all. On the contrary, negatively charged amino acids take part in repulsive electrostatic interactions, although repulsive interactions are weaker and not so common as interactions with positive amino acids. The highest repulsive interactions among amino acids from the binding site are observed for Asp334: Mg(H_2_O)_3_CTP1 (+71.2 kcal/mol) and Asp189: Mg(H_2_O)_3_CTP1 (+69.6 kcal/mol). For Asp334, the penetration is 2.7 kcal/mol, and for Asp189, it is equal to zero. For neutral polar amino acids, electrostatic interactions of Gln41 (−50.3 kcal/mol) and Tyr250 (−34.1 kcal/mol) with Mg(H_2_O)_3_CTP1 are noticeable. The side chain of Gln41 is arranged in a way to point the nitrogen atom toward oxygen atoms in triphosphate resulting in two HBs. Thus, penetration energy for this pair is −6.4 kcal/mol. Tyr250 creates one strong HB between the OH group from the side chain and triphosphate, and penetration energy is −9.4 kcal/mol. The interesting case is Glu33. Despite its negatively charged amino acid residue, the electrostatic interaction with Mg(H_2_O)_3_CTP1 is attractive (−11.4 kcal/mol). It interacts directly with the magnesium cation, not the nucleobase nor phosphate. Penetration energy of Glu33:Mg(H_2_O)_3_CTP1 is the highest for the whole complex and equals −65 kcal/mol. Glu33 creates three atom–atom contacts, two with the magnesium cation and one with a water molecule. One contact between Glu33 and the magnesium cation can be classified as a coordination bond.

**Table 3:** Electrostatic Interaction Energies Ees [kcal/mol] for Selected Amino Acid Residues of IFIT5 Interacting with Each of the Three Fragments of RNA

	Mg(H_2_O)_3_pppCCC			Mg(H_2_O)_3_pppUUU			Mg(H_2_O)_3_pppAAA		
residue	1	2	3	1	2	3	1	2	3
Glu33	–11.4	7.4	6.2	6.6	8.5	5.3	–5.4	8.6	5.8
Gln41	–50.3	–2.2	–1.4	–45.7	–2.7	–1.3	–57.0	–2.7	–1.4
Lys48	–133.1	–28.4	–17.8	–136.0	–29.6	–18.0	–131.7	–28.4	–18.1
Lys150	–214.6	–27.5	–19.5	–215.8	–31.3	–19.5	–216.2	–30.8	–20.3
Tyr185	–1.6	–5.8	0.4	–1.7	–6.9	0.4	2.4	0.9	3.0
Arg186	–159.7	–62.0	–26.1	–160.5	–60.1	–26.4	–160.5	–60.8	–27.8
Asp189	69.6	46.9	34.8	71.4	40.1	42.4	71.5	44.6	44.6
Tyr250	–34.1	1.0	0.6	–32.7	0.4	0.5	–30.0	0.8	0.7
Arg253	–176.1	–45.6	–27.2	–172.2	–48.1	–26.3	–170.1	–46.1	–26.9
Tyr254	–2.3	–26.5	–0.2	–2.3	–28.0	–1.1	–2.1	–31.0	–0.9
Lys257	–80.8	–67.6	–97.5	–81.8	–57.4	–94.1	–81.5	–65.4	–100.2
Arg294	–46.4	–22.7	–60.9	–47.0	–20.1	–53.6	–44.0	–20.3	–32.2
Asp334	71.2	14.4	23.8	72.7	28.5	22.7	69.0	23.4	23.2
Gln377	–4.5	–1.9	–3.9	–3.0	–1.0	–30.0	–2.5	–1.0	–3.2

For the second and third nucleotides, the observations found for the previously mentioned charged amino acids, apart from Glu33, are the same in nature, but energy values are generally smaller. Attractive electrostatic interactions have less negative energies, and repulsive interactions have less positive energies. Interactions of C2 or C3 with neutral Gln41 and Tyr250 are negligible. This is because these residues interact with the first RNA fragment by local hydrogens bonds which do not contribute to the interactions with the second and the third nucleotides. Also, the penetration energy for these interactions equals zero, which confirms their local nature. The new strong interaction is Tyr254:C2 (−26.5 kcal/mol). Tyr254 creates an HB with the C2 phosphate group; thus, also penetration energy is high (−9.6 kcal/mol) for this pair. Also, Lys257 interacts strongly with C2 (−67.6 kcal/mol) and C3 (−97.5 kcal/mol) by HBs with phosphate groups from both nucleotides. Glu33:C2 and Glu33:C3 interactions became slightly repulsive (7.4 and 6.2 kcal/mol, respectively), and penetration energy, in this case, is also zero.

**Figure 5:**
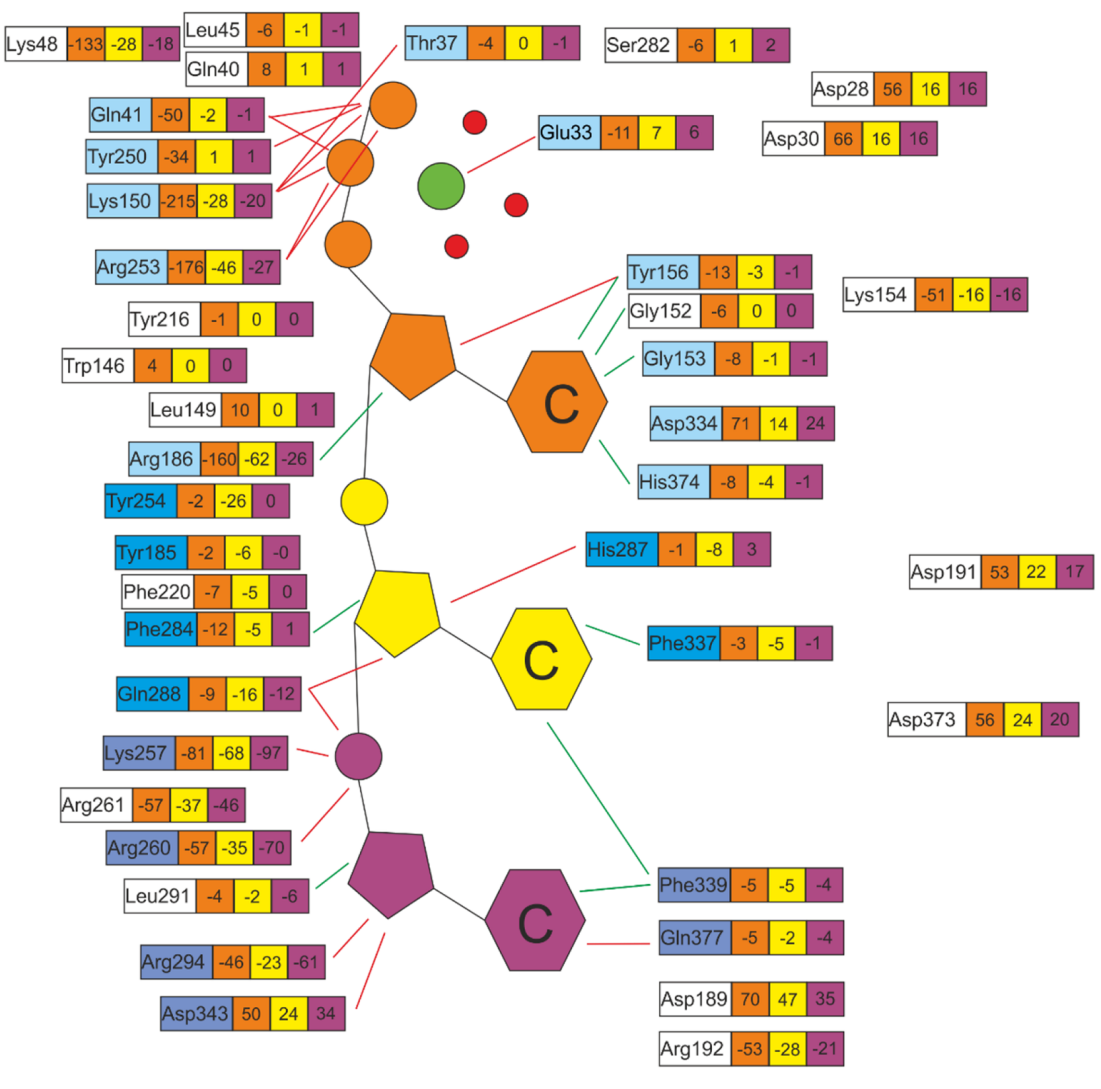
Detailed electrostatic interaction energies Ees [kcal/mol] between selected residues of IFIT5 and Mg(H_2_O)_3_pppCCC with separate contributions from each of the first three fragments marked orange, yellow, and violet, respectively. The first fragment of RNA contains also the magnesium cation (green circle) and three water molecules (red circles). Only residues within a 5 Å sphere around RNA and interaction energies over ±5 kcal/mol were considered. Labels of residues with a high contribution of penetration energy are marked blue. Contact lines are taken from structural analyses in the work of Abbas et al.^5^ for comparison, red lines indicate polar interactions, and green lines indicate van der Waals contacts.

**Figure 6:**
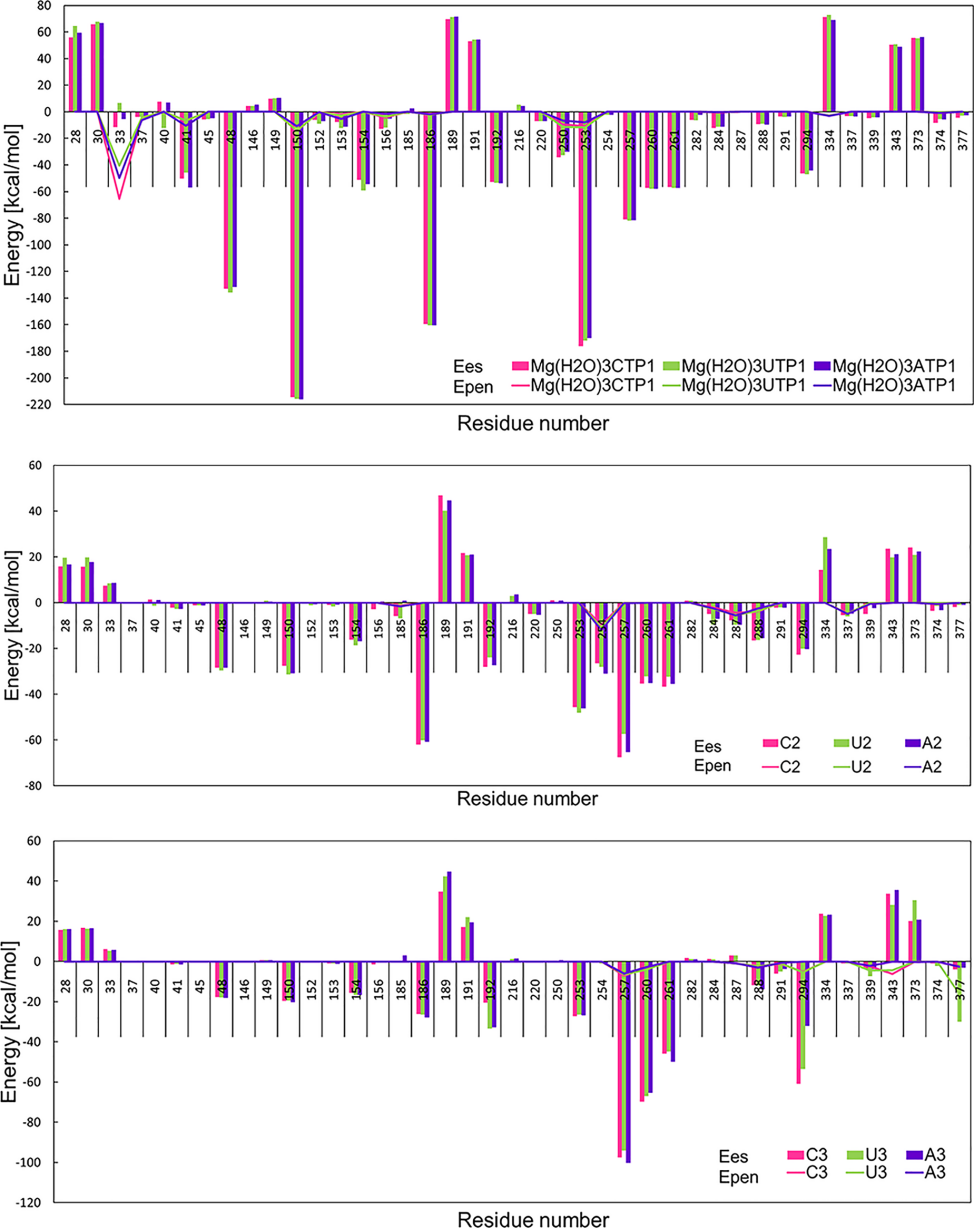
Electrostatic Ees and penetration Epen energy for the first, second, and third fragments of RNA interacting with selected amino acid residues of IFIT5.

Discussing penetration energy, which is always negative, the highest in absolute value is observed for the following residues: Glu33 (−65 kcal/mol) and then Lys150 (−14.2 kcal/mol), Arg253 (−10.7 kcal/mol), and Tyr250 (−9.4 kcal/mol) for interactions with Mg(H_2_O)_3_CTP1. Interactions with C2 Tyr254 (−9.6 kcal/mol) and C3 Lys257 (−7.2 kcal/mol) have the highest contribution to the penetration energy (Figures 5 and 6).

Interactions depicted in Figure 5 and in Figure S3 with red and green lines were taken from the work of Abbas et al.^5^ and found on the basis of the geometry analysis only. Thanks to our calculations, we were able to identify a larger amount of important interactions, which are shown without any lines. Abbas et al. in their work found 24 amino acids within a range of 4 Å for the IFIT5:Mg(H_2_O)_3_pppCCC complex. We chose different criteria. First, we found all amino acids which were within the range of 5 Å from Mg(H_2_O)_3_pppNNN molecules (where N is the C, U, or A nucleotide); there were 54 of them. By increasing the radius, we decreased the influence of geometry on our analysis. After that, we calculated electrostatic interaction energies Ees and set a filter to ±5 kcal/mol. It enabled us to distinguish 41 amino acids of particular importance. We confirmed that all the interactions found by Abbas et al. are significant. Moreover, we found other significant interactions: 17 new interactions for the IFIT5:Mg(H_2_O)_3_pppCCC complex. Our third filter was penetration energy Epen which helps to identify local interactions. We found three amino acids with high penetration energy (meaning interacting at a close distance), which were not found before: Tyr185, Tyr254, and Asp334. Electrostatic interactions for them are respectively nearly neutral for Tyr185, attractive for Tyr254, and strongly repulsive for Asp334. Besides, there are also long-range interactions found with our method with zero penetration contribution but with important electrostatic energies. The largest repulsive interaction found by us among charged amino acids is for Asp189 (151.3 kcal/mol, summed over the entire Mg(H_2_O)_3_pppCCC), the largest new attractive electrostatic interaction is for Arg192 (−101.5 kcal/mol), and among neutral amino acids, the strongest interaction (repulsive) is for Leu149 (10.6 kcal/mol, summed).

#### 3.4.2 The Complex of IFIT5 with Mg(H_2_O)_3_pppUUU

Similar to interactions of IFIT5 with pppCCC, interactions of IFIT5 with pppUUU are the strongest for positively charged amino acids situated in the binding site. These interaction are the strongest again for the first RNA fragment (Mg(H_2_O)_3_UTP1) and weaker but still remarkable for U2 and U3. For example interactions, Ees energy of Mg(H_2_O)_3_UTP1 with Lys150 is −216 kcal/mol, when Lys150:U2 is −31 kcal/mol and Lys150:U3 is −19 kcal/mol. For negatively charged amino acids, the trend is the same. Contrary to Mg(H_2_O)_3_CTP1 (and to Mg(H_2_O)_3_ATP1) which interactions with Glu33 are attractive, the Glu33:Mg(H_2_O)_3_UTP1 interaction is repulsive (6.6 kcal/mol). It may be caused by replacing the original sodium cation in the deposited 4HOS structure for magnesium. One distinguishable important unique feature for the pppUUU is a strong interaction of Gln377:U3 (−30 kcal/mol), which is caused by double hydrogen bond. For Gln377:C3 and Gln377:A3, this interaction is much weaker, −4 kcal/mol and −3 kcal/mol, respectively, as only one HB is observed.

Abbas et al. in their work found 23 amino acids interacting with the pppUUU complex (Figure S3a). They observed an interaction with Asp334, which they omitted for pppCCC and pppAAA complexes. We showed that this interaction is important in all three complexes. On the contrary, for pppUUU, they did not choose His374 and Arg192, whereas they listed both of them for both pppCCC and pppAAA complexes. They claim that Arg192 interacts with the fourth nucleotide, which is missing in the IFIT5:pppUUU structure, but our calculations show that Arg192 has strong electrostatic interactions also with the first, second, and third nucleotide and thus should not be neglected. Again, we showed that these two interactions His374 and Arg192 are important in all three complexes.

#### 3.4.3 The Complex of IFIT5 with Mg(H_2_O)_3_pppAAA

Although the third complex of IFIT5 contains RNA built with a purine: adenine, instead of previously being built with pyrimidine, the interactions do not differ much (Figure 6). Strong interactions with positively charged amino acids have similar energy values. It is caused by the fact that for an attractive interaction it is a negatively charged phosphate that is mostly responsible for electrostatic interaction, not the nucleobase. What is interesting about the complex of IFIT5-Mg(H_2_O)_3_pppAAA is that interactions for Tyr185:Mg(H_2_O)_3_ATP1 and Tyr185:A2 are slightly repulsive (+2 kcal/mol and +1 kcal/mol), whereas for Mg(H_2_O)_3_pppCCC and Mg(H_2_O)_3_pppUUU, analogous interactions are attractive. The residue Tyr185 interacts with Mg(H_2_O)_3_CTP1 and Mg(H_2_O)_3_UTP1 with the energies equal to −2/–2 kcal/mol with C2 and U2 equal to −6/–7 kcal/mol. This is caused by the different orientations of the hydrogen atom from the hydroxyl group of the Tyr185 side chain in complex with adenine. In IFIT5:Mg(H_2_O)_3_pppCCC and IFIT5:Mg(H_2_O)_3_pppUUU complexes, this hydrogen atom is directed toward phosphate in RNA, and in the IFIT5:Mg(H_2_O)_3_pppAAA complex, it points in the opposite direction (toward Arg261). Also, interaction with Arg294:A3 is twice as weak as Arg294:C3 and Arg294:U3. This is the result of the longer distance between the shortest non-hydrogen atoms in Arg294 and A3 which is equal to 4.3 Å, whereas for Arg294:C3 and Arg294:U3, it is 2.9 Å. The different values of the interaction energy between Gln41 and the first RNA fragment are a result of different donor–acceptor distances.

Abbas et al. in their work found 24 amino acids interacting with the pppAAA molecule (Figure S3b). They observed the interaction with Asp189 as the interaction with the fourth nucleotide. We showed that this interaction is important in all three complexes with all of the first, second, and third RNA fragments.

### 3.5 Dynamics of IFIT5 in Complex with RNA

The fully atomistic molecular dynamics simulations of the IFIT5 protein and its complexes give insight into the dynamics of the complex. First, we aimed to investigate the stability of the RNA nucleotides in the binding site. For this purpose, we have extended the RNA up to 12 adenines to obtain the IFIT5-ppp12A complex. We have chosen this complex because the multiadenine RNA chains are commonly seen in biological systems and experimental setups. The analysis of the B-factor values for this complex derived from simulations (Figure S4) shows that the first four nucleotides are less mobile in comparison with other nucleotides in the RNA chain.

The complex of IFIT5 with ppp5C contains two conformations of the second nucleobase, *syn* and *anti*. We have decided to check the behavior of this nucleobase in solution in the IFIT5-ppp5C complex. The torsion angle O4′–C1′–N1–C2 distributions and changes in time of the second nucleotide in two sets of simulations of the IFIT5-ppp5C complex are shown in Figure S5. It is visible that the simulations that started from the IFIT5-ppp5C-(C2*syn*) conformation switch to the *anti* conformation within the first 200 ns of the simulation. On the other hand, the simulations starting from the IFIT5-ppp5C-(C2*anti*) system stay in the *anti* conformation. Nevertheless, the RNA is overall relatively stable in the binding site (Figure S6). Similarly as in the IFIT5-ppp12A complex, here also the positions of the first four nucleotides are more stable than the other nucleotides in the complex.

For the IFIT5-ppp12A, IFIT5-ppp5C(C2*syn*), and IFIT5-ppp5C(C2*anti*) systems, for which we have performed the simulations, we are able to follow the distances between the centers of mass of chosen amino acids and nucleotides in time. There is a clear correlation between the large in absolute value electrostatic interaction energies within the binding site shown in Table 3 and the averaged distances between the residues during the simulation gathered in Table S4. The most attractive electrostatic interactions were observed for Lys150 and the first RNA fragment. In the simulations, the average distance between those two residues, Lys150 and CTP1, was very short, around 0.80 nm in all analyzed systems. In addition, the distance was not changing much during the simulation as evidenced by small standard deviations, up to 0.21 nm. The second and third strongest electrostatic attractive interactions were visible between the first RNA fragment and Arg253 and Arg186, which is in agreement with the short average distances visible in the simulations, ranging from 0.63 to 0.97 nm, and small distance variations, ranging from 0.05 to 0.12 nm. The residues with the strongest repulsive interactions with the first RNA fragment were Asp334 and Asp189, for which the average distances varied from 1.06 to 1.28 nm, and the standard deviations were between 0.11 and 0.21 nm.

For the IFIT5-ppp5C(C2*syn*) and IFIT5-ppp5C(C2*anti*) systems, for which we have performed the simulations, we calculated the UBDB+EPMM electrostatic interaction energies to investigate the variation of the electrostatic energy in these complexes. To do a fair comparison, as a reference we took energies computed for the crystal structure with the C2 residue in its *anti* configuration. The difference between energies for C2*syn* and C2*anti* crystal structures is not very large, 26 kcal/mol in total in favor for the C2*syn*, and is located mostly only on a few amino acid residues (Lys150, Arg253, Asp334, Figure S7). In general, mean values of electrostatic energies obtained for six structures representing results from the molecular dynamics simulations are similar to that computed on the basis of the single crystal structure (Figure S8). There were only a few amino acids for which the difference between energy from the crystal structure and the mean energy from molecular dynamics was larger than 20 kcal/mol: Glu33, Gln41, Lys48, Lys150, Asp191, Arg192, Lys257, and Gln288. For most of them, their sample standard deviations were also high; hence, the differences are not so meaningful. Only for three residues with high difference in energy, the difference is larger than their three sample standard deviations: Gln41, Lys48, and Gln288. In fact, sample standard deviations for many amino acid residues were quite high. For the majority of the residues, their standard deviations are at the level of 10% of their mean interaction energy. Almost 60 of them had deviations larger than 5 kcal/mol. Among them, there were many amino acid residues from the binding site which strongly interact with the RNA. The largest variation in energy was observed for Lys150 and Arg186, with sample standard deviations equal to 56 and 51 kcal/mol, respectively. This suggests that the energy for a single frame structure may not correspond well with the energy of the crystal structure. Only analyses for the whole assembly of structures from molecular dynamics are valid if one wants to contrast them with the crystal structure. In fact, the crystal structure is also representing a protein structure averaged through time (the time of the measurement) and space (many copies in one single crystal).

The mean value of the electrostatic interaction energy for the whole protein interacting with the RNA equals −778 kcal/mol, and the sample standard deviation is 110 kcal/mol. The value is smaller than for the crystal structure (−975 kcal/mol for C2*anti* and −1001 kcal/mol for C2*syn*), though it is still within two sample standard deviations. Obtained fluctuation in total electrostatic energy indirectly confirms that the difference in total energy observed for crystal structures with various sequences is not significant, and electrostatic interactions of RNA to IFIT5 are not sensitive to the RNA sequence.

We have compared the electrostatic interactions calculated with the UBDB+EPMM method and the simple point charges using the same six structures from molecular dynamics simulations as above. The results are shown in Figure S9. The major difference relates to the local interactions with the magnesium cation, visible, for example, in the interactions with Glu33. In the UBDB+EPMM method, Glu33 interacts with the whole Mg(H_2_O)_3_pppCCC chain with the mean electrostatic energy of interactions equal to −32 kcal/mol and the sample standard deviation of 14 kcal/mol. On the other hand, the electrostatic interaction energy estimated with the point charges derived from the CHARMM36m force field and the TIP3P water model was on average above 79 kcal/mol with the sample standard deviation 23 kcal/mol. Moreover, the energies of the interactions between the phosphate groups and amino acid residues are visibly different when estimated by using the UBDB+EPMM method and the point charges from the force fields (see residues Lys257 and Arg260). Even though many contributions to the electrostatic energy are similar for both methods, the UBDB+EPMM method shows larger variability in the electrostatic energy for the short-range interactions, giving better balanced and richer information than the simple point charge method.^54^ It is also very well visible for the change in balance in electrostatic interactions of negatively charged, positively charged, and neutral amino acid residues. The net energy of electrostatic interactions for charged residues interacting with the Mg(H_2_O)_3_pppC1 fragments of the RNA is ca. 10% larger in absolute value for the UBDB+EPMM method than for point charges, while for interactions with the C2 and C3 fragments, the net energies are almost the same (Table 4). The largest difference in the net energies is visible for neutral amino acid residues, for which the net energy from the UBDB+EPMM method is ca. 75% larger in absolute value than from the point charges.

**Table 4:** Mean Values of Electrostatic Interactions Energies [kcal/mol] Calculated on the Basis of Six Molecular Dynamics Simulation Runs with the UBDB+EPMM Method or with Point Charges for the All Positively Charged, Negatively Charged, and Neutral Amino Acid Residues Interacting with Each Fragment of Mg(H_2_O)_3_pppCCC ((1-Mg(H_2_O)_3_pppC1, 2-C2, and 3-C3)

	Mg(H_2_O)_3_pppCCC			
	1	2	3	sum
	UBDB+EPMM			
positively charged	–2781	–1048	–1091	–4920
negatively charged	2462	931	886	4280
neutral	–93	–26	–18	–138
	point charges			
positively charged	–2557	–1053	–1078	–4688
negatively charged	2224	934	884	4041
neutral	–53	–16	–10	–79

We have also investigated the influence of the presence of RNA on the dynamics of the IFIT5 protein. For this purpose, we have performed the simulations of the ligand-free IFIT5 in two protonation states of Asp334, as the p*K*_a_ of this amino acid was close to pH 7.2. The simulations show that the protonation of this residue did not have a meaningful impact on the dynamics of the system (see Figure S10). However, the presence of RNA changes the global movement of the subdomains in IFIT5 around the pivot subdomains. This movement can be effectively measured using a pseudodihedral angle consisting of the centers of mass of subdomain I, subdomain II, pivot, and subdomain III. The analysis has shown that this pseudodihedral angle in IFIT5 without ligands stays within 60–70 degrees, whereas in the presence of RNA it shifts to around 80 degrees (Figure S11). The local fluctuations shown in Figures S4, S6, and S10 suggest that the presence of RNA is stabilizing the protein. In the complexes, also increased mobility of the flap containing residues Arg192 and Glu193 is observed. We noticed that this flap frequently remains closed in the presence of RNA, whereas it is open and less mobile in IFIT5 without the ligands.

### 3.6 IFIT5 Binding Affinity

**Figure 7:**
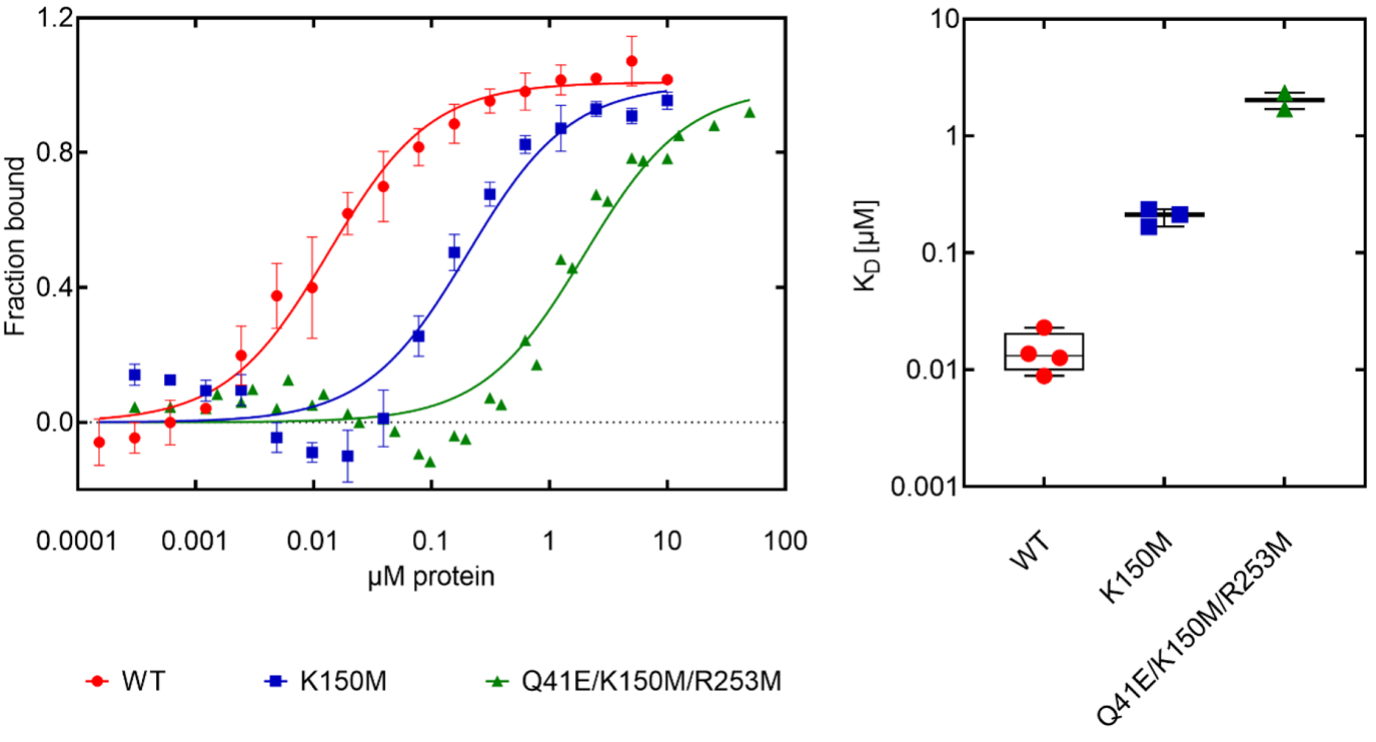
Binding of IFIT5 WT (apparent K_D_ = 13.45 ± 1.29 nM), K150M (apparent K_D_ = 204.1 ± 33.69 nM), and Q41E/K150M/R253M (apparent K_D_ = 1.98 ± 0.36 μM) to 5′-ppp-AAAAAGGAAGGUCy5 measured by Microscale Thermophoresis (MST). Data were analyzed with Graphpad using the one-site specific binding model, and the apparent K_D_ values are reported as mean ± SEM.

Several groups tried to measure the binding affinity of the IFIT5 protein with pppRNA. In 2013, Abbas et al.^5^ estimated it as 250–500 nM by the EMSA method. The following year Kumar et al.^8^ using the primer extension method calculated it as 372 (±21) nM. More recent studies by Miedziak et al.^7^ showed that the binding affinity of IFIT5 with pppRNA is equal to 42.7 (±1.6) or 113 (±12) nM depending on the presence of the magnesium cation, whereas binding to GpppRNA or m^7^GpppRNA is weak (Table 5). Seeing inaccuracy in experimental results, we decided to try another method to estimate binding affinity: microscale thermophoresis (MST). Our results show (Figure 7) that wild type IFIT5 binds with RNA with an apparent K_D_ = 13.45 ± 1.29 nM. Then, we prepared two mutants of IFIT5: K150M and the triple mutant Q41E/K150M/R253M and measured their binding affinity. The results were as follows: an apparent K_D_ = 204.1 ± 33.69 nM for K150M and an apparent K_D_ = 1.98 ± 0.36 μM for Q41E/K150M/R253M, respectively.

**Table 5:** Experimental Binding Affinities K_D_ [nM] of Selected Complexes of IFIT5-RNA Complexes

		effect	binding affinities K_D_ [nM]				
variant	subdomain	a	b	c	d	e	f
WT			1.4	250–500	372 (21)	113 (12) with Mg	13.45 (1.29)
						42.7 (1.6) no Mg	
E33A	I	low	3				
E33A/D334A	I		2				
T37V	I	high					
T37A	I		0.9				
Q41E	I	high	2				
Q41E/K150M		high					
Q41E/K150M/R253M		high					1980 (360)
K150M	II	high	100				204.1 (33.69)
Y156F	II	neutral	4				
R186H	II		3				
R186A	II	high					
Y250F	II	high	2				
R253M	II	high	30				
Y254F	II	high	2				
R260E	II	high					
H287A	pivot	low					
Q288E	pivot	high	3				
L291A	pivot				1		
R307A	pivot				1		
D334A					2		
F337A		high					
F339A	III				3		
K415A	III				3		
K426A	III				2		

a Abbas et al.,^5^ Method pulldown, buffer 50 mM Tris, pH 7.5, 100 mM NaCl, 5% (v/v) glycerol, 0.2% (v/v) Nonidet-P40, 1.5 mM MgCl_2_, pppRNA7SK-as 378 nt (with GAA as the first three nucleotides). For this column, the effect of particular mutations on binding affinity is described.

b Katibah et al.,^9^ Method EMSA, buffer 20 mM Tris·HCl, pH 8.0, 200 mM NaCl, 5 mM MgCl_2_, 10% (v/v) glycerol, 2 mM DTT, and 0.1 mg/mL BSA, pppRNA WNV 30nt (with AGU as the first three nucleotides).

c Abbas et al.,^5^ Method EMSA, buffer 10 mM Tris pH 7.9, 100 mM NaCl, 1 mM TCEP, 5% v/v glycerol, pppRNA 44nt (with GGG as the first three nucleotides).

d Kumar et al.,^8^ Method Primer extension, buffer 20 mM Tris, pH 7.5, 100 mM KCl, 2.5 mM MgCl_2_, 1 mM ATP, 0.2 mM GTP, 1 mM DTT and 0.25 mM spermidine, pppRNA β-globin (with GAC as the first three nucleotides).

e Miedziak et al.,^7^ Method Biolayer Interferometry, buffer 50 mM phosphate buffer pH 7.2, 150 mM NaCl, 10% glycerol, 0.5 mM DTT, 0.1% BSA, and 0.05% Tween 20, pppRNA 16nt (with GGG as the first three nucleotides).

f Our experiment, Method microscale thermophoresis, PBS, 5% glycerol, 0.5 mM TCEP, 0.05% Tween20, 1 mM MgCl2, pppRNA-cy5 12nt (with AAA as the first three nucleotides).

We decided to compare the experimental results for our mutants to the previous experiments, as IFIT5 mutants have been proposed and investigated since the structure of IFIT5 was described.^5,7−9,55,56^ Moreover, thanks to our calculations, it is now possible to compare mutants selected on the basis of structural studies only to the results based on electrostatic energy computations. There were different research groups that analyzed binding abilities of the IFIT5 protein (wt and mutants) with different RNAs by various biophysical methods, and the data are shown in Table 5. Binding buffers, in general, were similar, though they differed in ionic strength (100 vs 200 mM NaCl) or concentration of magnesium ions. Also analyzed RNA had different lengths and sequences.

We can distinguish two kinds of protein regions on which authors focused. First, it is the pocket where the 5′-end of RNA is bound, mainly amino acids interacting with phosphate or the triphosphate group or the magnesium cation. Here, e.g., E33A, T37V/A, and Q41E mutants were constructed. The results obtained for those residues are debatable. For E33A substitution, both Abbas et al.^5^ and Katibah et al.^9^ showed only a weak influence on the binding of RNA to IFIT5. However, for T37A and Q41E, Katibah et al. showed a weak influence on binding, whereas for both T37V and Q41E, Abbas et al. showed a strong influence on binding strength. Our calculations show that E33 does not have a significant value of electrostatic interaction energy; however, the penetration contribution is significant. T37 interacts electrostatically weakly, whereas Q41 interacts strongly, and both have a noticeable penetration contribution. Moreover, the nucleotide with adenine at the first positions has a slightly larger penetration contribution to the interaction than with cytidine and uracil. Unfortunately, in all biophysics experiments, RNA used contained A or G as the first nucleotide and not C or U, so we could not compare our theoretical calculations directly with them.

The second group of amino acids is positively charged amino acids, lysines and arginines, situated along the RNA binding site. They were mutated to neutral amino acids alanine or methionine, e.g., K150M, R186A, and R253M. Results of all research groups agree with the statement that mutations of many lysine and arginine residues have a strong impact on the binding affinity of IFIT5; however, for the R186 residue, the Katibah et al. group shows that although R186H strongly compromised IFIT5 binding to 5′-p WNV30 RNA and cellular RNA, this substitution did not affect IFIT5 binding to 5′-ppp WNV30 RNA by EMSA. Our calculations confirm that these residues are important (including R186), and we showed high electrostatic interaction energies for lysine or arginine residues interacting with the first RNA fragment. K150 was the first strongest attractively interacting amino acid both with Mg(H_2_O)_3_N1 and with the sum of the three fragments of RNA, while R253 was the second and R186 was the third. K150 and R253 belong also to the residues for which the highest penetration contribution to the energy is observed. Also, our MST experiment confirms that the K150M mutant of IFIT5 has a strong influence on binding affinity to RNA, and the triple mutant Q41E/K150M/R253M influences it even much stronger.

## 4 Conclusions

We have shown that with the use of the UBDB+EPMM method it is possible to describe electrostatic interactions not only in a qualitative but also in a quantitative way. Small changes in the orientation of molecules or even positions of a few atoms can influence the electrostatic interaction energy, which is distinguishable by our method (vide Tyr185). Moreover, we found strong long-distance interactions which have not been distinguished on the basis of geometry only in the article of Abbas et al. Looking at the penetration contribution, it was possible to find all amino acids with strong local interactions. With our calculations, we can define the binding site without using the distance criteria. However, using distance criteria (5 Å) helps us to limit amino acids with long-distance interactions to those belonging to the binding site, creating a better-defined binding site. Combining three filters used by us (5 Å range, ±5 kcal/mol of interaction energy, and strong penetration), we were able to find the well-defined binding site of investigated complexes.

Even though in investigated complexes the geometries of some atoms differ slightly causing small differences in interaction energies, the total electrostatic interaction energy of the three complexes is comparable. Moreover, contributions of particular amino acid residues to the electrostatic interactions with the RNA in the three complexes are comparable. Thus, we confirmed that the electrostatic energy of IFIT5 interaction with pppRNA does not depend on the sequence of the RNA.

## Supporting Information Available

Figures S1–S11, Tables S1–S4, verification of magnesium-related atom types; conformations of cytidine in 4HOR structure; detailed electrostatic interaction energies for 4HOS and 4HOT complexes; results from molecular dynamics simulations; positions of added hydrogen atoms in water molecules; and average distances between the centers of mass of chosen amino acids and nucleotides from simulations (PDF)
Excel file with electrostatic interaction energies calculated with UBDB+EPMM method or point charges method for all amino acid residues for crystal structures and representative structures from six molecular dynamics simulation runs (ZIP)
