# Supplementary material for: The Role of Electrostatic Interactions in IFIT5-RNA Complexes Predicted by the UBDB+EPMM Method
Source: J Phys Chem B. 2022 Nov 3;126(45):9152–67. doi: 10.1021/acs.jpcb.2c04519 (PMC9677429; doi:10.1021/acs.jpcb.2c04519)
Supplement: Supplementary file 1 — jp2c04519_si_001.pdf [file jp2c04519_si_001.pdf]

Supporting Information

The Role of Electrostatic Interactions in IFIT5-RNA Complexes Predicted by the UBDB+EPMM Method

Urszula Anna Budniak[1], Natalia Katarzyna Karolak[1,2], Marta Kulik[1], Krzysztof Młynarczyk[1], Maria Wiktoria Górna[1], Paulina Maria Dominiak[1]**

[1]Biological and Chemical Research Centre, Department of Chemistry, University of Warsaw, ul. Żwirki i Wigury 101, 02-089 Warszawa, Poland

[2]Nencki Institute of Experimental Biology, Polish Academy of Sciences, ul. Ludwika Pasteura 3, 02-093 Warszawa, Poland

*Correspondence e-mail: mgorna@chem.uw.edu.pl, pdomin@chem.uw.edu.pl

Verification of magnesium-related atom types

Calculations for new magnesium-related atom types were based on the structures deposited in two databases: Organic Structure Database (CSD) and Inorganic Crystal Structure Database (ICSD). Structures should be reliable to be used in calculations, thus some conditions must be fulfilled: R factor below 5%, structures without disorder, measured at low temperature. Following structures from CSD were selected: GEQBIO, BELYEY and BELYAU. All of them are organic crystals containing magnesium coordinated by six ligands including two or three water molecules. From ISCD following structures were used for calculations: 2549, 2550, 187, 188, which contain Mg coordinated by six water molecules. Structures were refined using LSDB and XD2016 programs. Refinement was conducted up to full convergence of parameters: Pval, Kappa and multipoles up to hexadecapoles. Kappa' for magnesium hasn't been refined because it has not converged to physical values. To verify newly introduced atom types connected with magnesium cation, intermolecular electrostatic energy calculations were performed for selected dimers of small molecules extracted from the GEQBIO structure deposited in CSD (Figure S1). The dimers included: two dimers built from two anions of diaqua-bis(hydrogen diphosphato)-magnesium and two dimers built from one diaqua-bis(hydrogen diphosphato)-magnesium anion and one bis(Ethane-1,2-diammonium) cation.

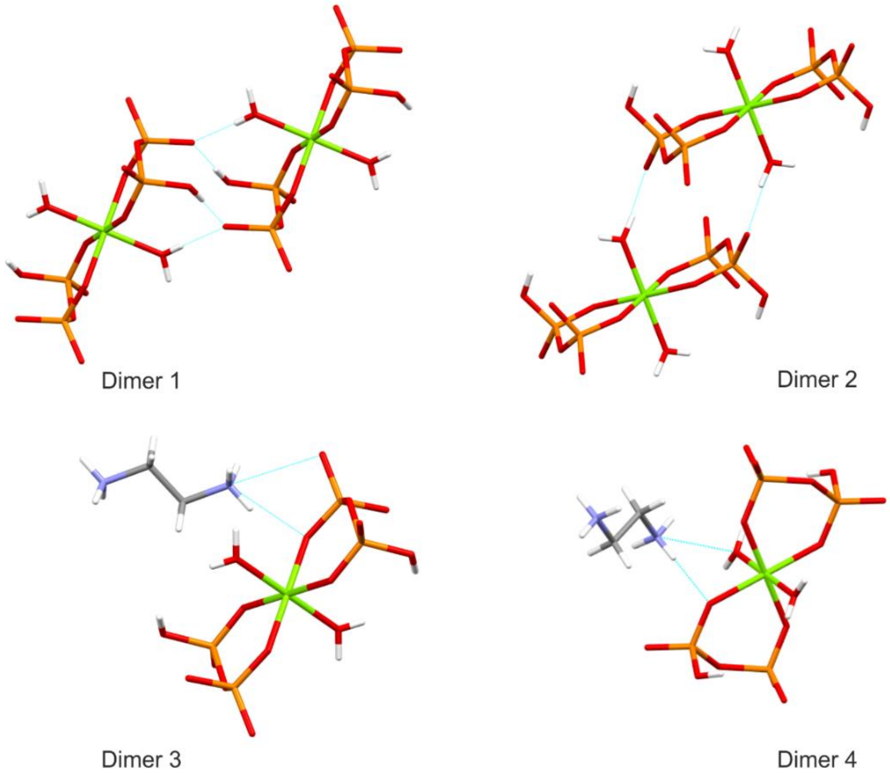

Figure S1. Geometries of four dimers extracted from the GEQBIO structure selected for energy calculations.

The Ees energies from the UBDB models of electron density were calculated using the EPMM method implemented in the XDPROP module of the XD2016 package. Several values for numerical integration parameters were checked. Default integration parameters in xd.mas are: iqt 2 Nrad 50 Nang 194. We checked the combination of iqt 2 or 3, Nrad 50 or 99 and Nang 194, 590 and 1202 for integration in XD.

The UBDB+EPMM energies were then compared with the corresponding reference results obtained directly from the molecular wavefunctions at the same level of theory (B3LYP) and the same basis sets (6-31G**) which is applied to build the UBDB. The *SPDFG* program [Volkov, A., King, H. F. & Coppens, P. (2006). J. Chem. Theory Comput. 2, 81–89.] was used to compute the reference energies. The E_{es} values obtained from *SPDFG* were taken as reference points as they have excellent agreement with $E_{Pol}(1)$ obtained from the Symmetry-

Adapted Perturbation Theory based on Density Function Theory (DFT-SAPT) [Kumar et al. *J. Chem. Theory Comput.* 2014, 10, 4, 1652–1664] [Williams, H. L.; Chabalowski, C. F. J. Phys. Chem. A 2001, 105, 646– 659] [Jansen, G.; Hesselmann, A. J. Phys. Chem. A 2001, 105, 11156– 11157].

Table S1. Interaction energy [kcal/mol] of two dimers computed with the UBDB+EMPP method with different variants of integration parameters compared to the reference method.

			Dimer1	Dimer2
ref			542	634
UBDB+EMMP				
Iqt	Nrad	Nang		
2	50	194	568	648
2	99	590	565	648
2	99	1202	567	648
3	99	590	565	648
3	99	1202	567	648

The UBDB+EPMM energies closest to the reference were obtained for iqt=2, Nrad=99 and Nang=590 (Table S1). Also with this set of integration parameters, the time of calculations was still acceptable. The results show (Table S2) that energies obtained from the UBDB+EPMM method were close enough to the reference energies, within the errors of the method.

Table S2. Comparison of interaction energy [kcal/mol] of four dimers computed with the UBDB+EPMM method (iqt 2 Nrad 99 Nang 590) to the reference method.

	Dimer1	Dimer2	Dimer 3	Dimer 4
ref	542	634	-479	-445
UBDB+EPMM	565	648	-486	-449

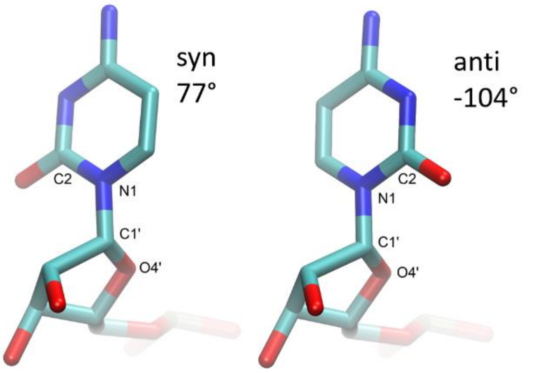

Figure S2. The torsion angle O4'-C1'-N1-C2 defines the *syn* and *anti* conformations of cytidine in the crystal structure 4HOR [Abbas, Y.M. *et al*. (2013) Nature, 494:60-64].

a)

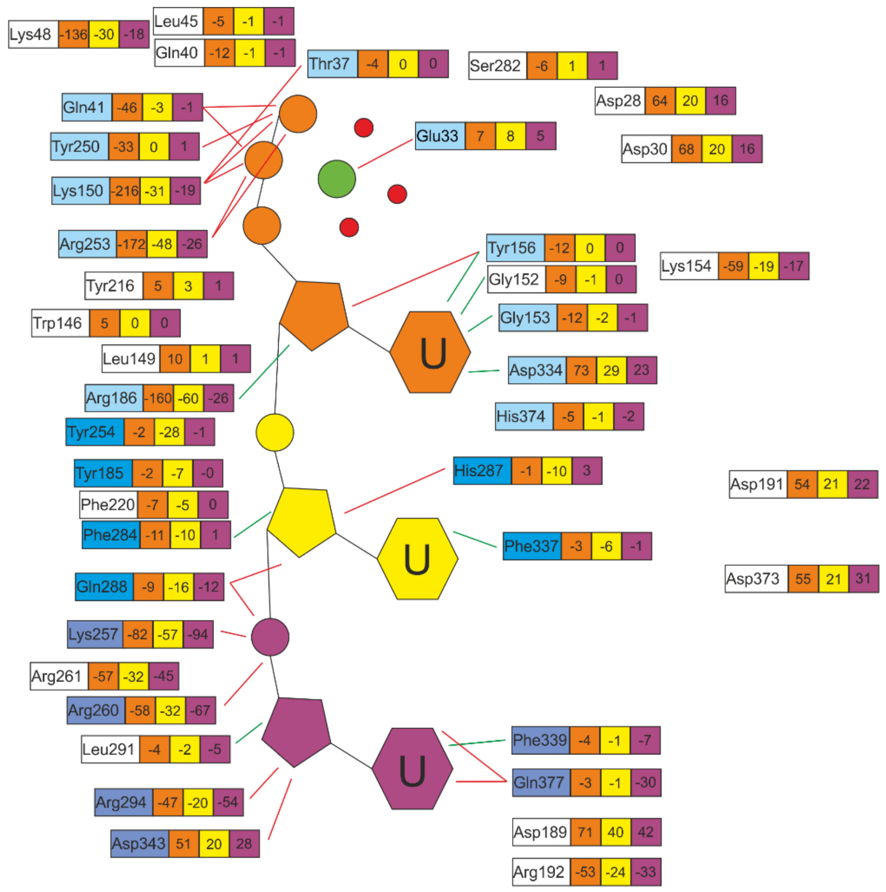

b)

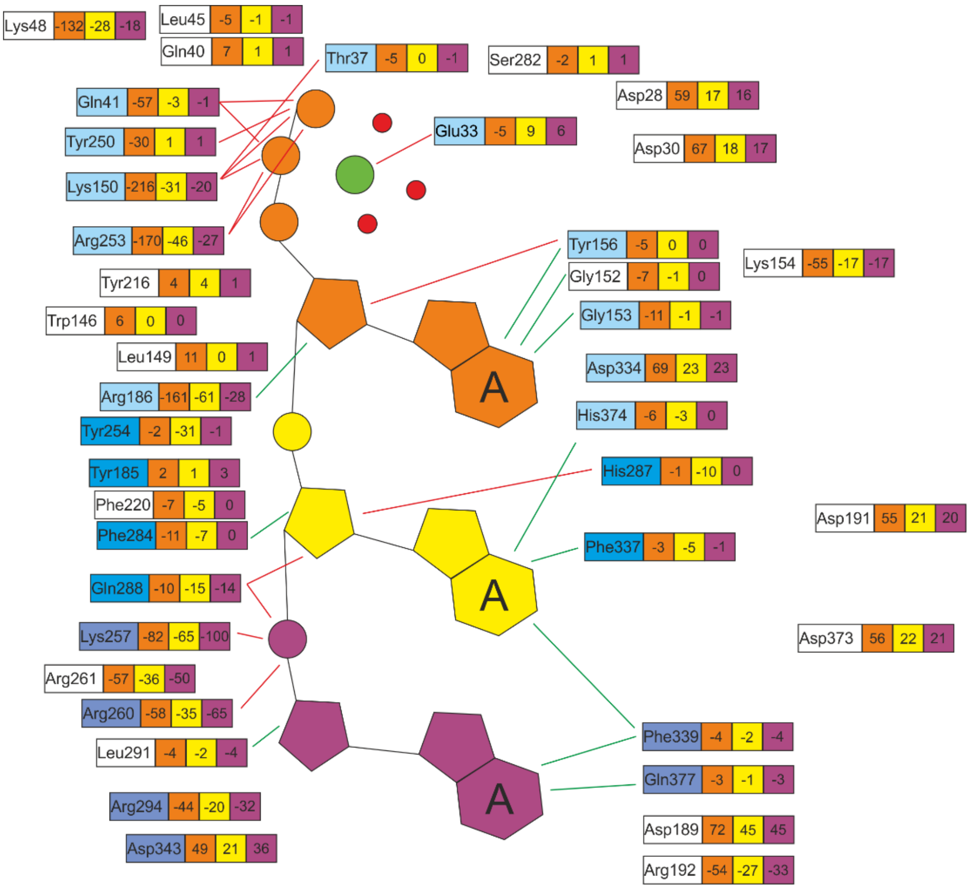

Figure S3. Detailed electrostatic interaction energies between selected residues of IFIT5 and oligo-RNA a) pppUUU and b) pppAAA with separate contributions from each of the three first nucleotides marked orange, yellow, and violet, respectively. The first fragment of RNA contains also magnesium cation (green circle) and three water molecules (red circles). Only residues within a 5A sphere around oligo-RNA and interaction energies over 5 kcal/mol were considered. Labels of residues with a high contribution of penetration energy are marked blue. Contact lines are taken from structural analyses in the work of Abbas, Y.M. *et al*. (2013) Nature, 494:60-64 for comparison, red lines indicate polar interactions, and green lines Van der Waals contacts.

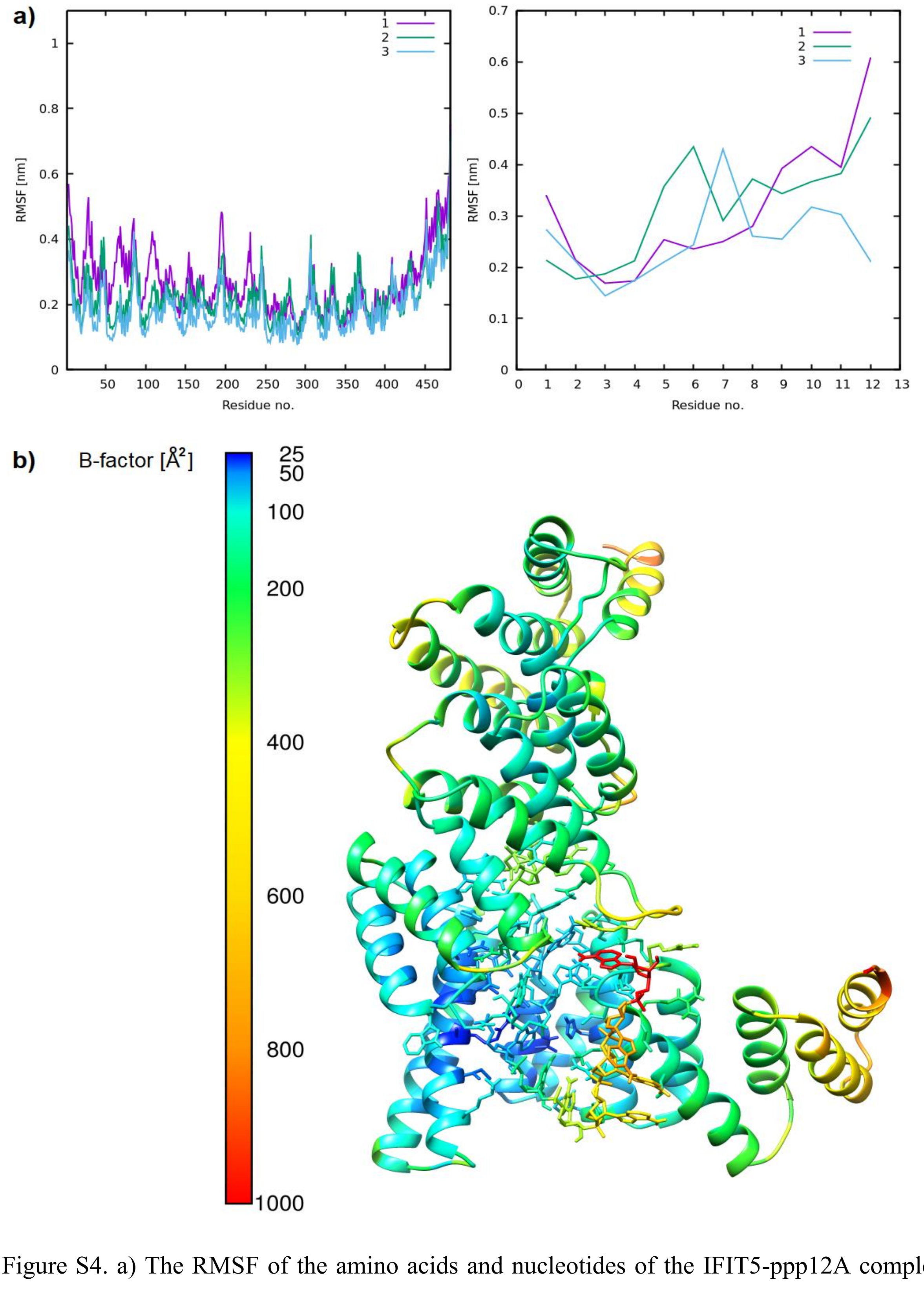

Figure S4. a) The RMSF of the amino acids and nucleotides of the IFIT5-ppp12A complex, calculated for the last 500 ns of all simulations (runs 1 to 3). b) The IFIT5-ppp12A complex structure, colored according to the B-factor of each residue for the first run. The B-factor values are calculated based on the RMSF values in Gromacs.

a)

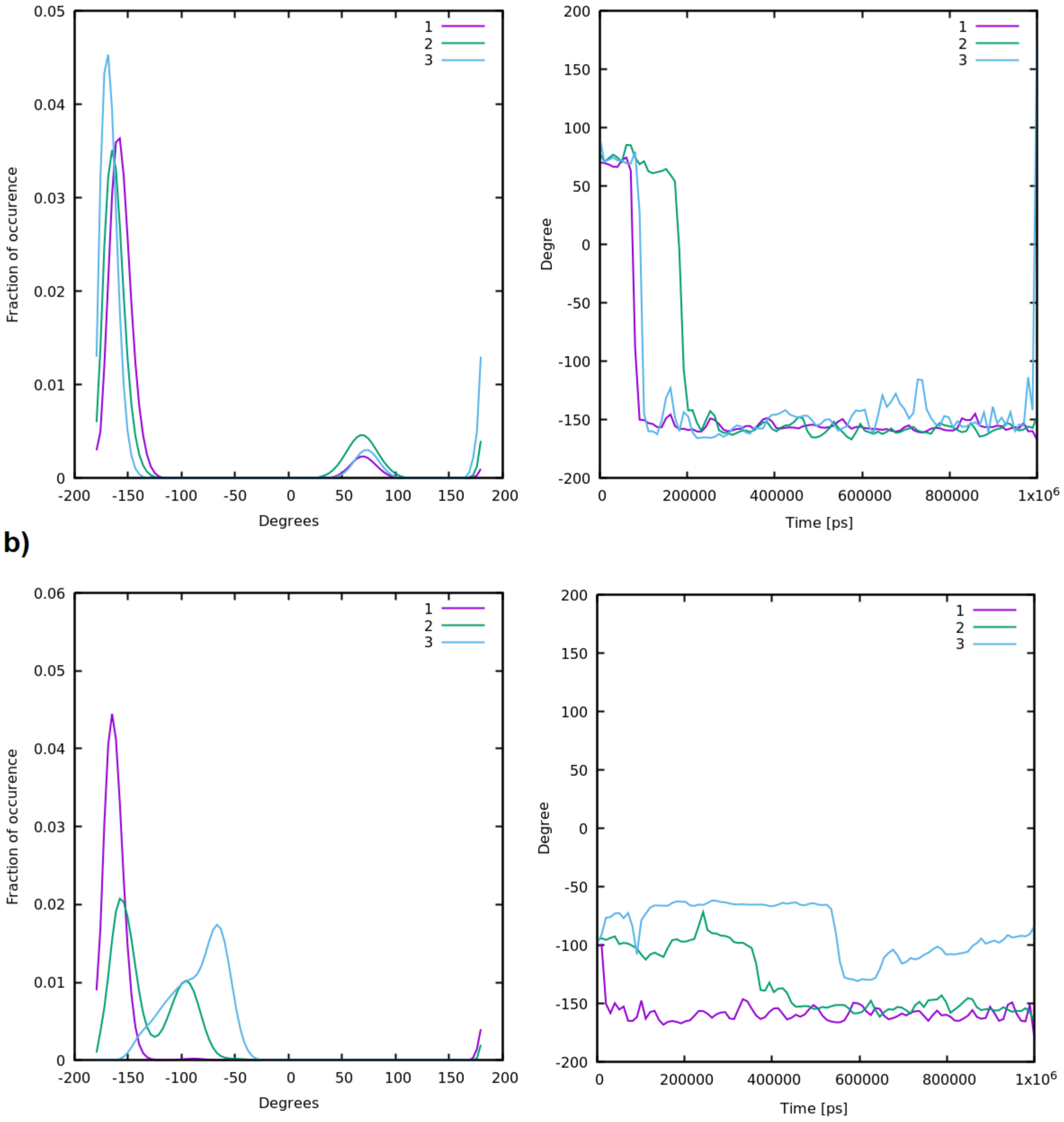

Figure S5. The torsion angle O4'-C1'-N1-C2 distributions and changes in time of the second nucleotide in two sets of three independent simulations of the IFIT5 with ppp5C complex, starting from a) *syn* and b) *anti* conformations of C2.

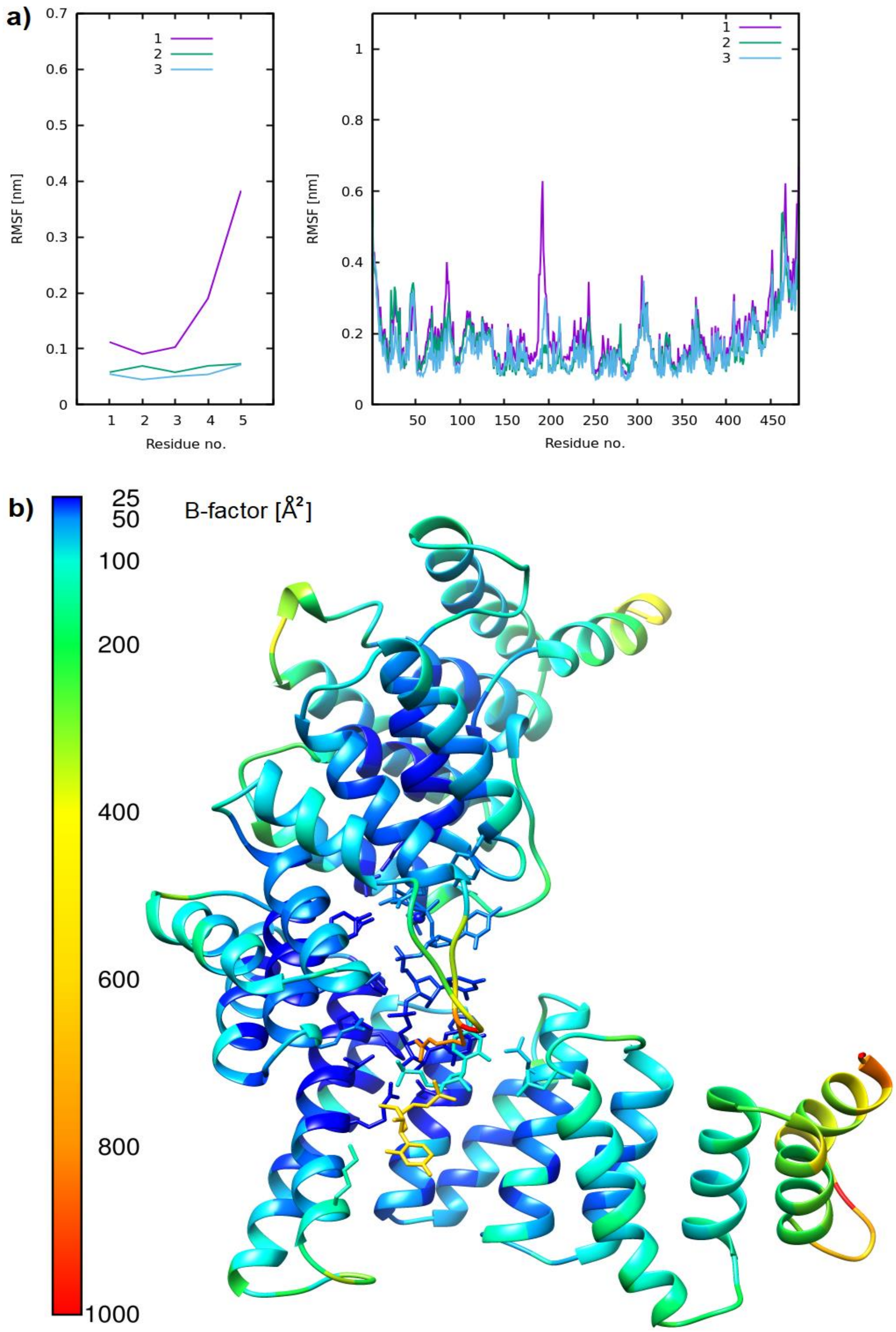

Figure S6. The RMSF of the amino acids and nucleotides of the IFIT5-ppp5C complex, calculated for the last 500 ns of all simulations (runs 1 to 3), starting from a) *syn* and c) *anti* conformations of C2. The IFIT5-ppp5C complex structure, colored according to the B-factor of each residue, for b) the first run of the simulation starting from *syn* conformation and d) the second run of the simulation starting from *anti* conformations of C2.

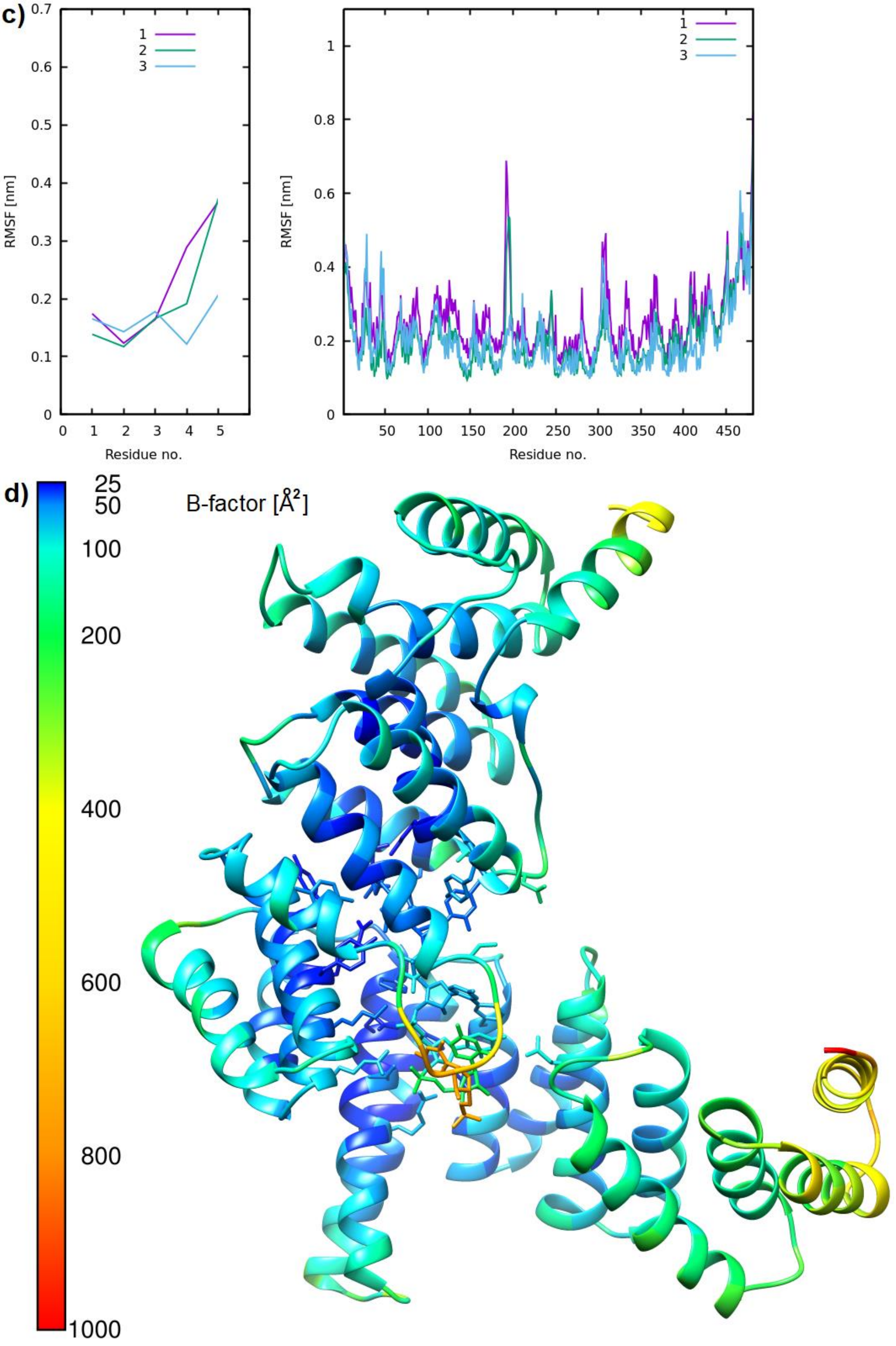

Figure S6. (continued)

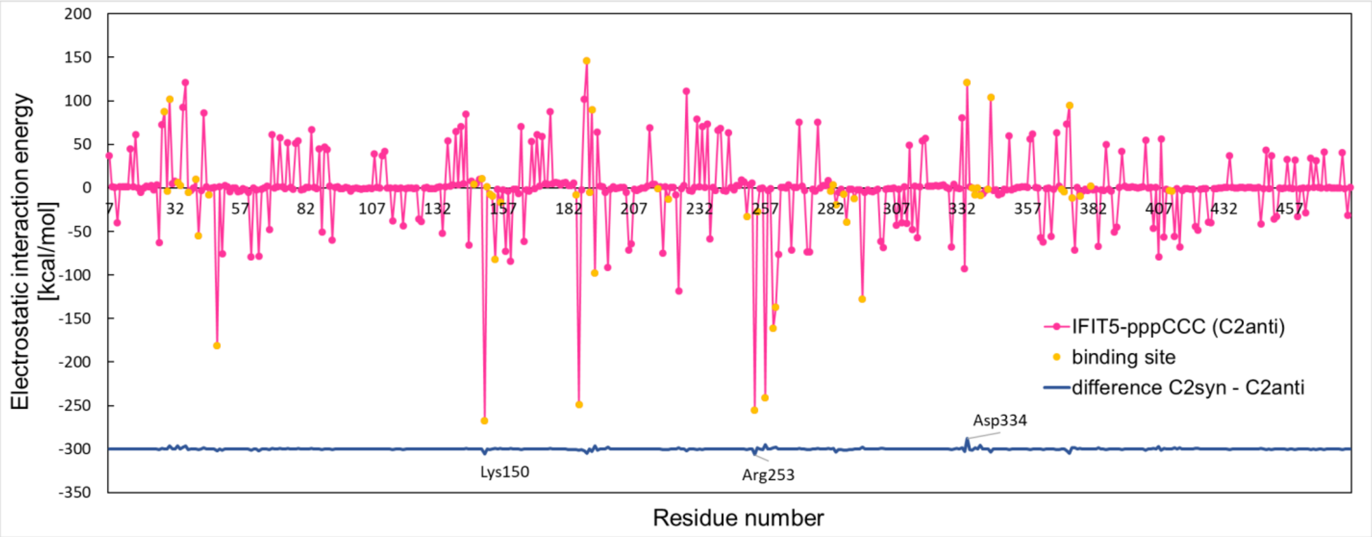

Figure S7. Electrostatic interaction energies Ees [kcal/mol] for particular amino acid residues of IFIT5 protein interacting with the whole $Mg(H_2O)_3$pppCCC chain (from the 4HOR crystal structure, C2*anti*) marked by magenta line together with amino acid residues which are situated in the binding site marked by orange. The dark blue line indicates the difference between the electrostatic interaction energies for C2*syn* and C2*anti* crystal structures [kcal/mol] shifted by -300 kcal/mol for better visibility.

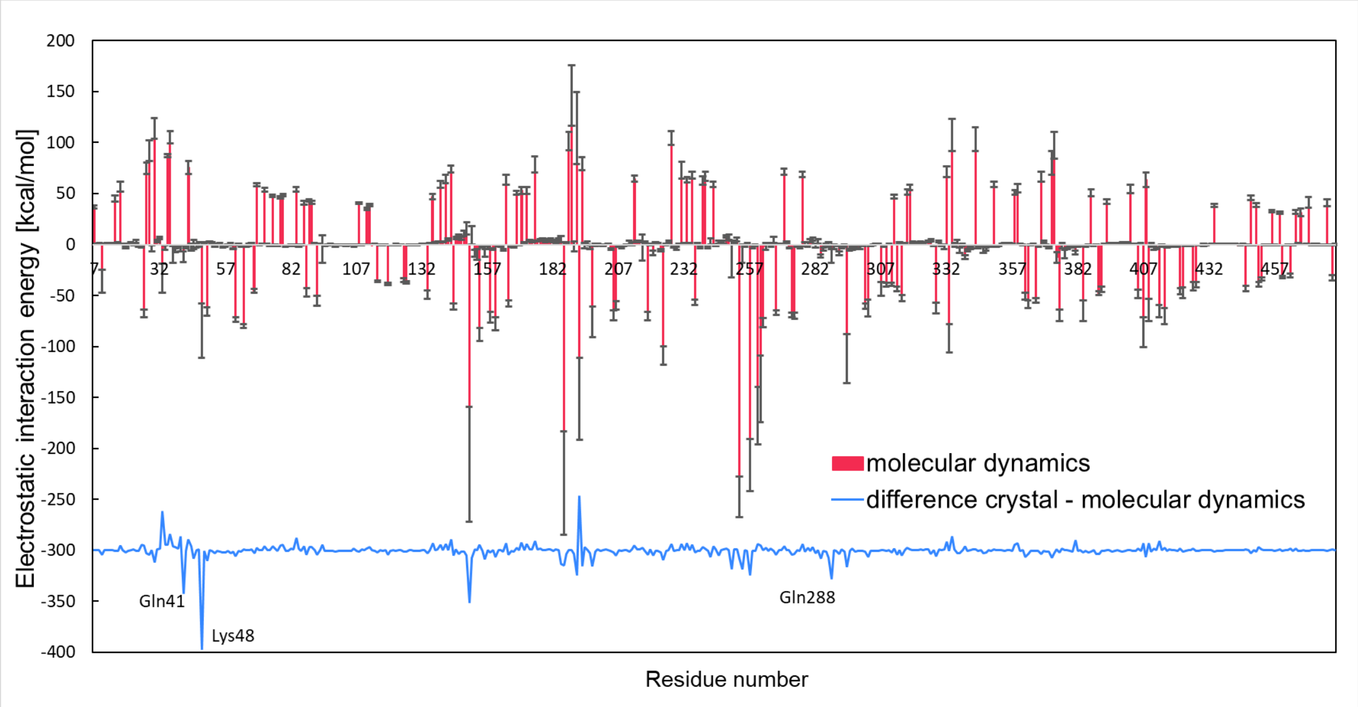

Figure S8. Mean values of the electrostatic energies Ees [kcal/mol] for interactions between particular amino acid residues of IFIT5 protein and the whole $Mg(H_2O)_3pppCCC$ chain calculated with the UBDB+EPMM method on the basis of six molecular dynamics simulation runs (red lines). The sample standard deviations are shown with black bars. The blue line pictures the difference between the electrostatic interaction energies computed on the basis of the 4HOR crystal structure (C2*anti*) and mean electrostatic energies from molecular dynamics [kcal/mol] shifted by -300 kcal/mol for better visibility.

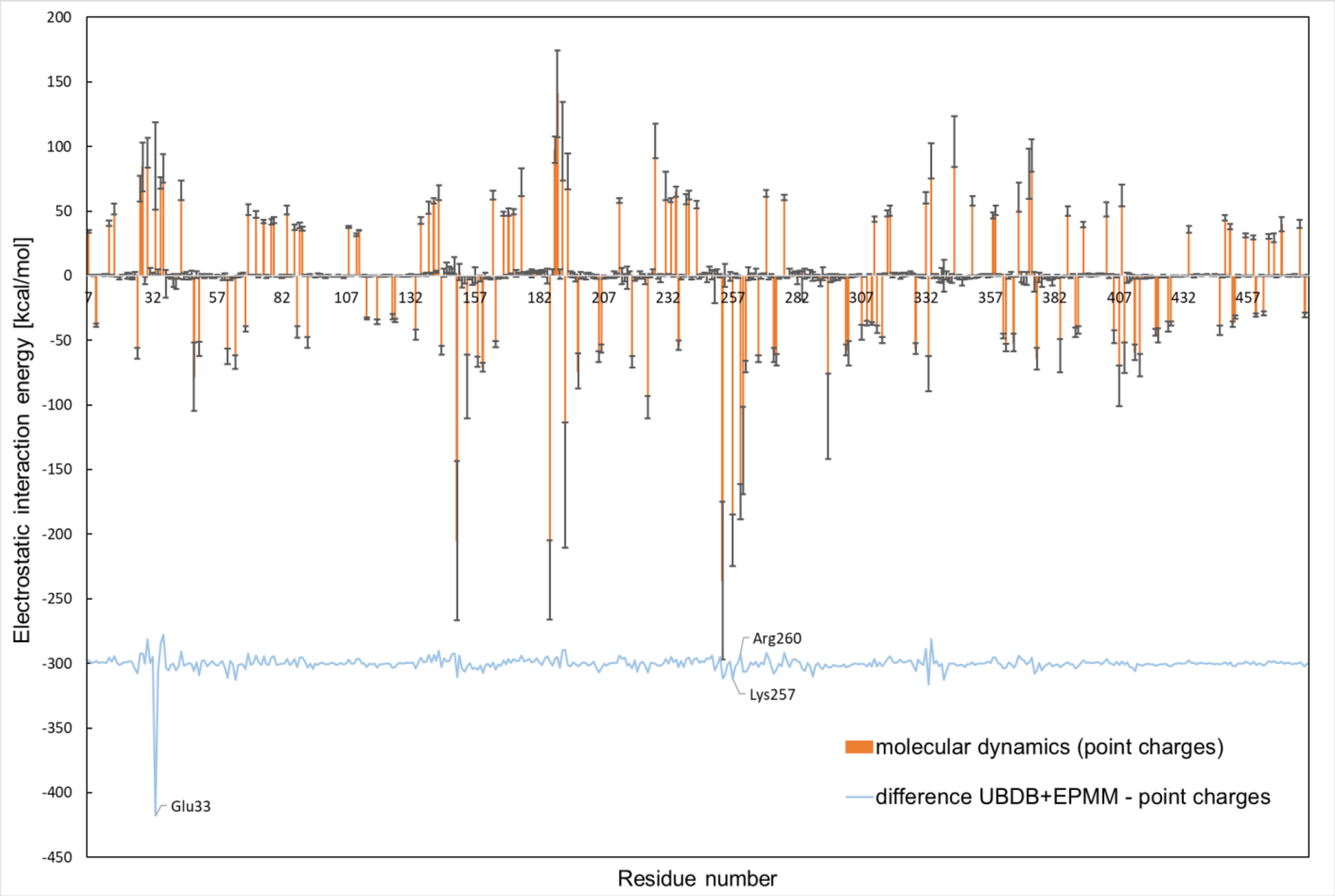

Figure S9. Mean values of the electrostatic energies Ees [kcal/mol] for interactions between particular amino acid residues of IFIT5 protein and the whole $Mg(H_2O)_3$pppCCC chain calculated with the simple point charges on the basis of six molecular dynamics simulation runs (orange lines). The sample standard deviations are shown with black bars. The light blue line pictures the difference between the mean electrostatic interaction energies computed for the same molecular dynamics structures with the UBDB+EPMM method and the simple point charges shifted by -300 kcal/mol for better visibility.

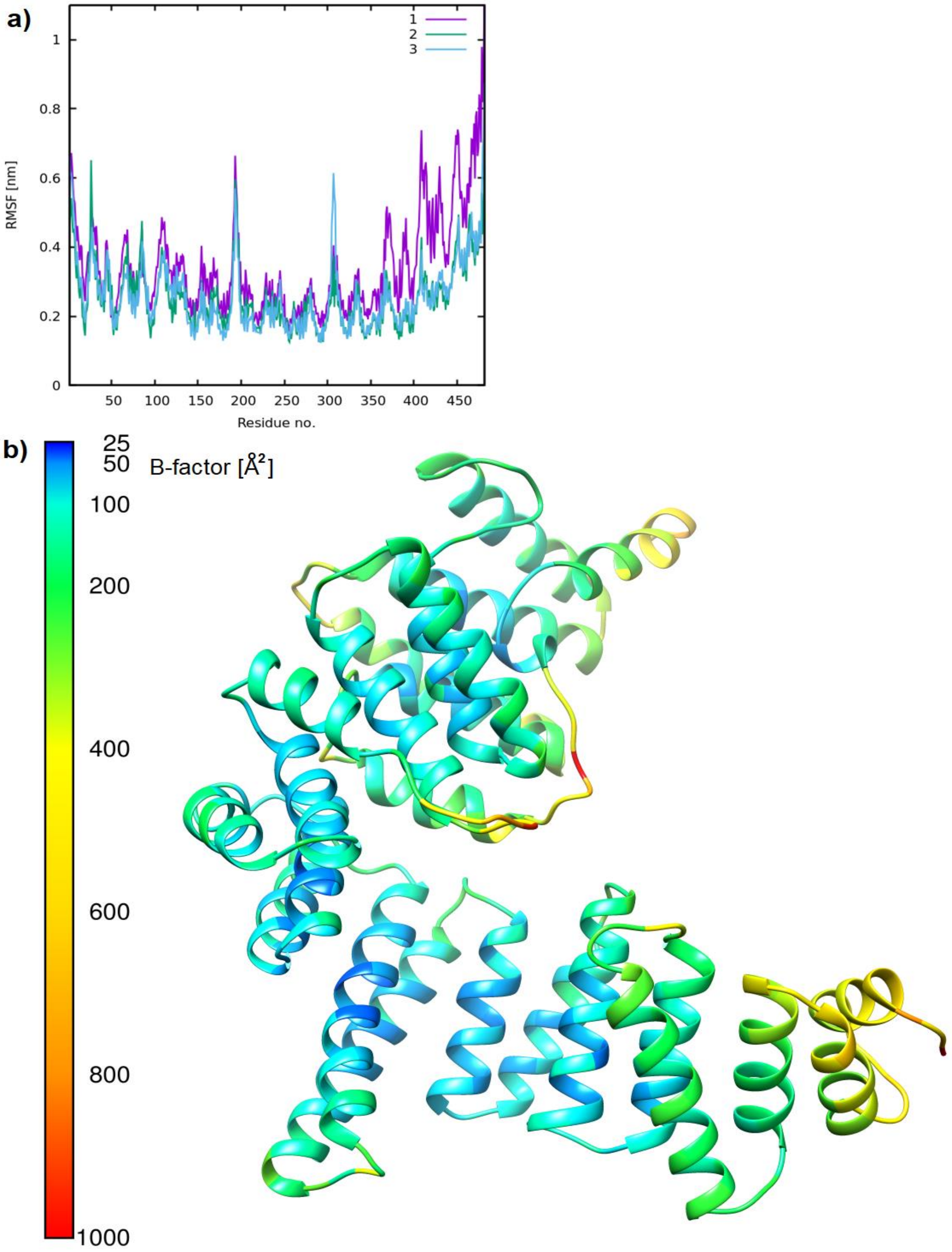

Figure S10. The RMSF of the amino acids of the IFIT5 structure without ligands, calculated for the last 500 ns of all simulations (runs 1 to 3), a) with and c) without the protonation of Asp334. The IFIT5 structure, colored according to the B-factor of each residue, b) for the second run of the simulation with the protonation of Asp334 and d) for the first run of the simulation without the protonation of Asp334.

c)

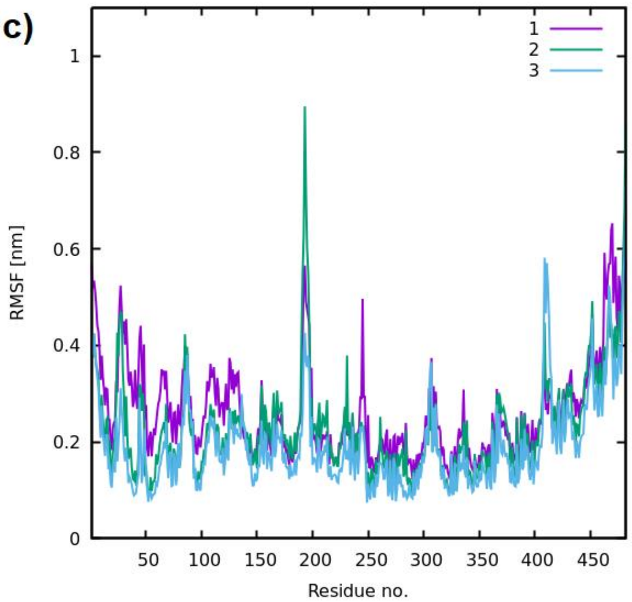

d)

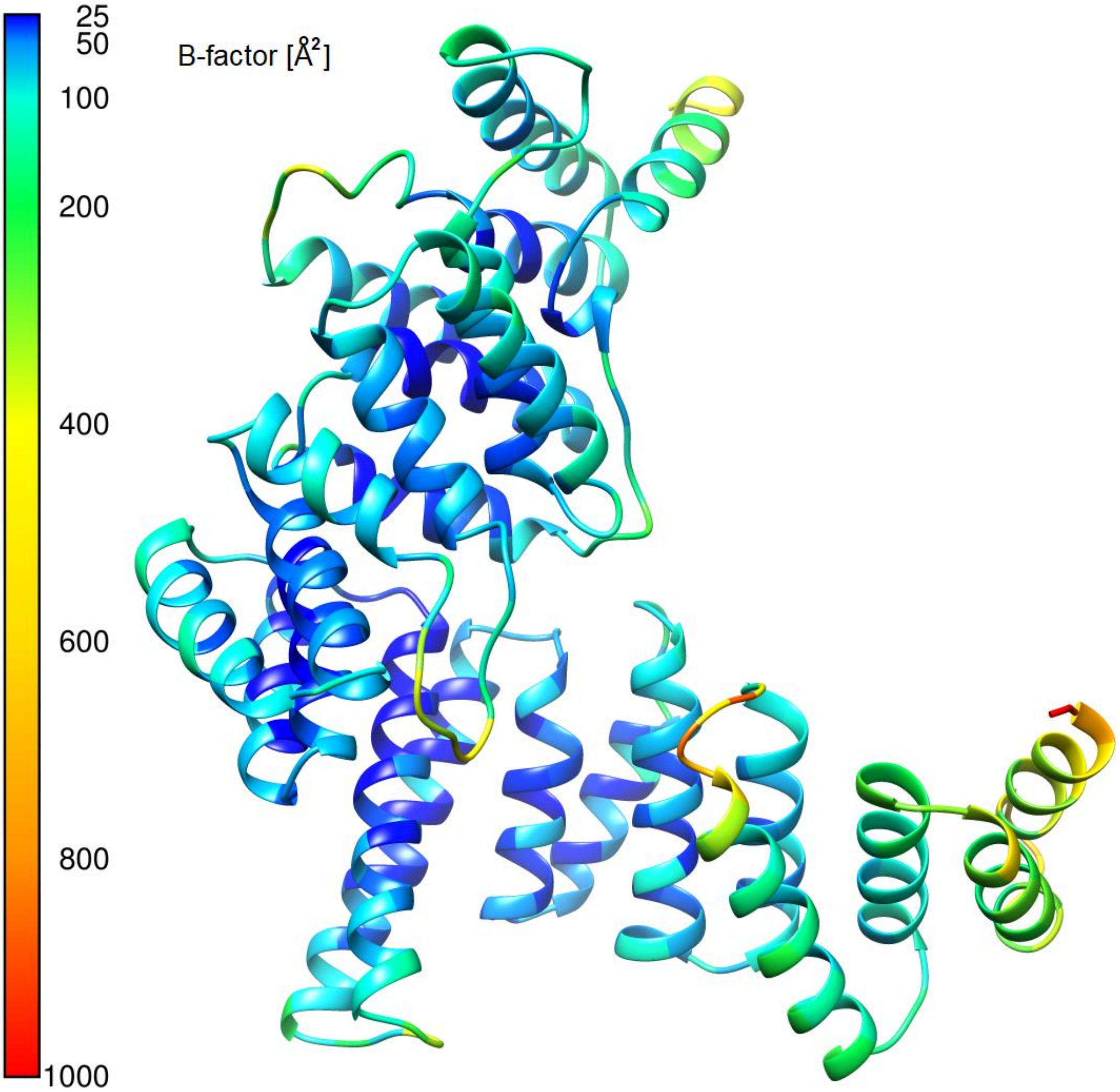

Figure S10. (continued)

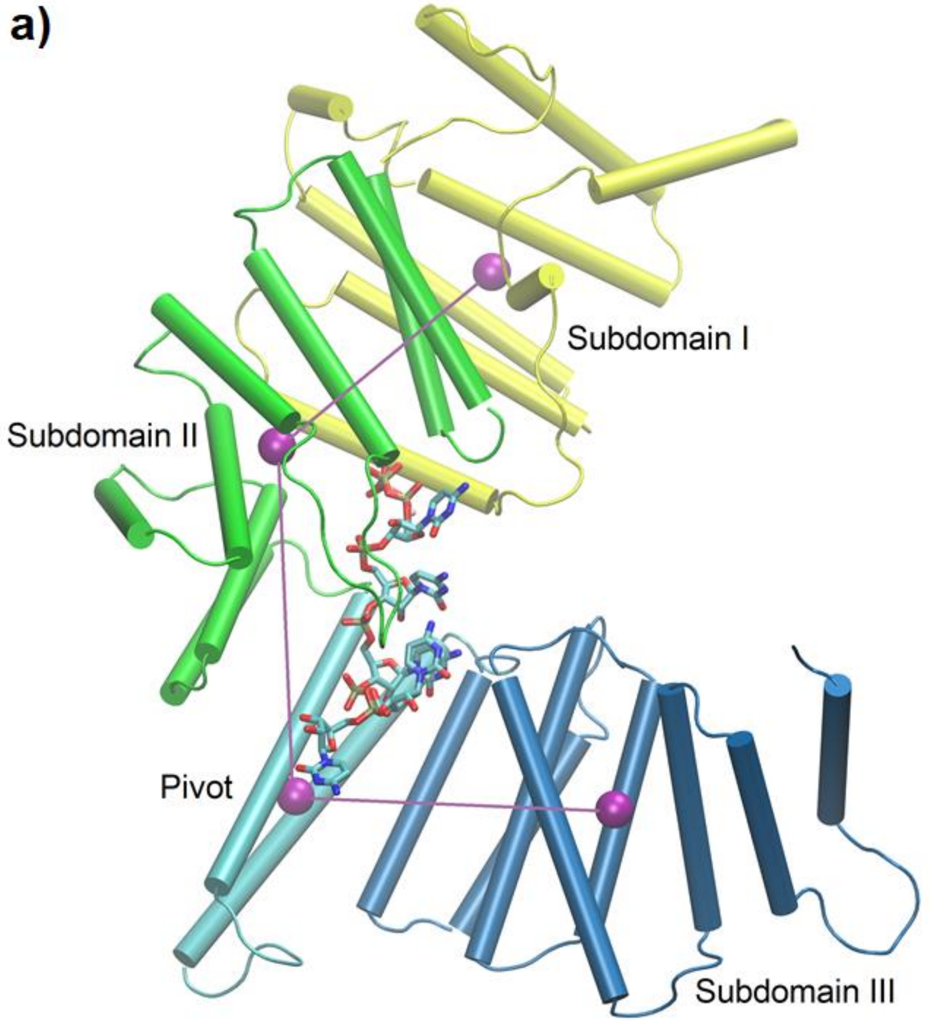

Figure S11. The changes of the pseudodihedral angle in time and pseudodihedral angle distribution of IFIT5 with and without ligands. a) The pseudodihedral angle is defined as the center of mass of Subdomain I, Subdomain II, Pivot, and Subdomain III, as defined by Abbas, Y.M. *et al.* (2013) Nature, 494:60-64. The changes of the pseudodihedral angle in time and the pseudodihedral angle distribution for three independent simulation runs for the systems: b) IFIT5-ppp12A complex, c) IFIT5-ppp5C complex starting from the *syn* conformation of C2, d) IFIT5-ppp5C complex starting from the *anti* conformation of C2, e) IFIT5 structure without ligands and without the protonation of Asp334, f) IFIT5 structure without ligands and with the protonation of Asp334.

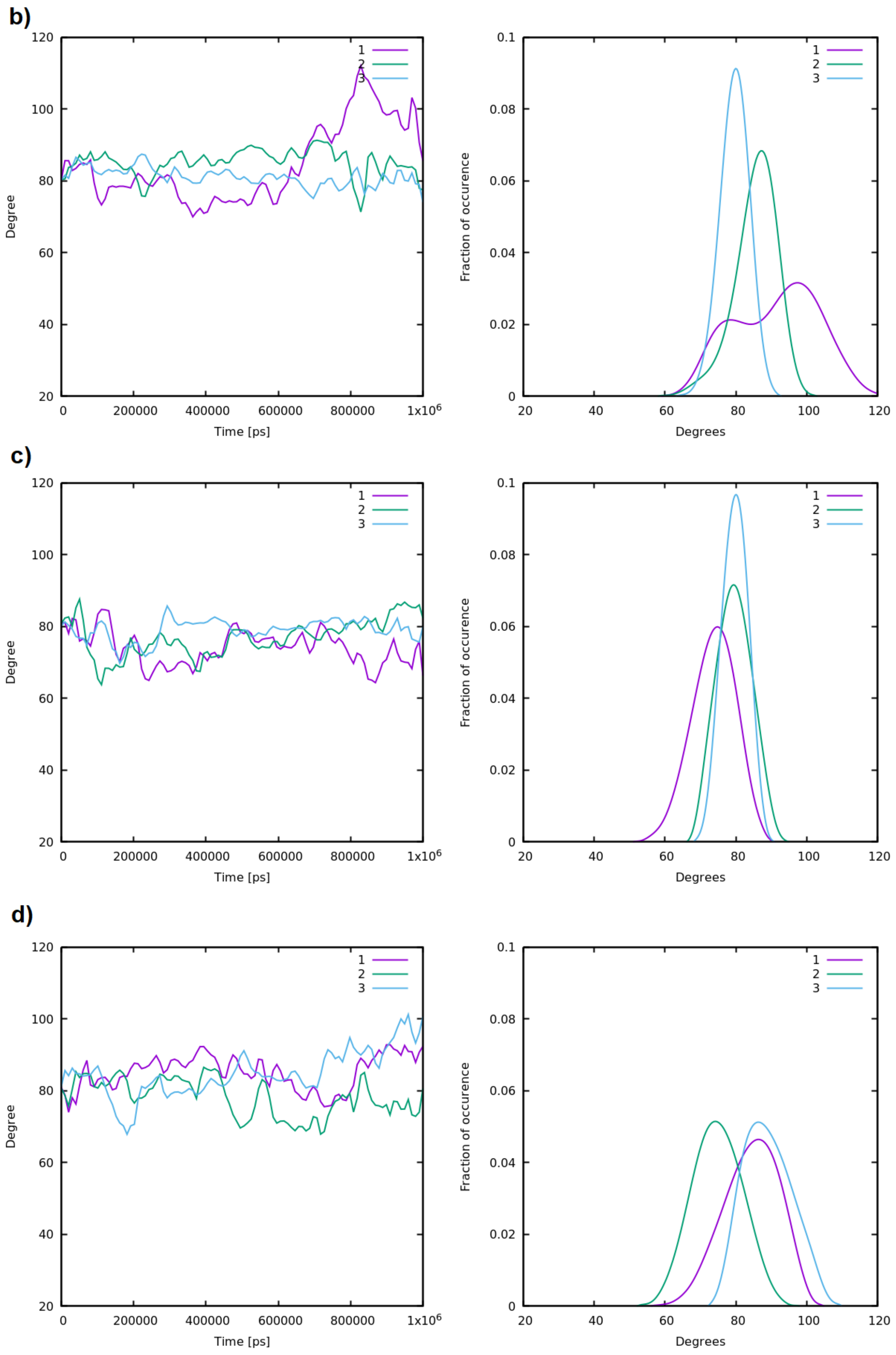

Figure S11. (continued)

e)

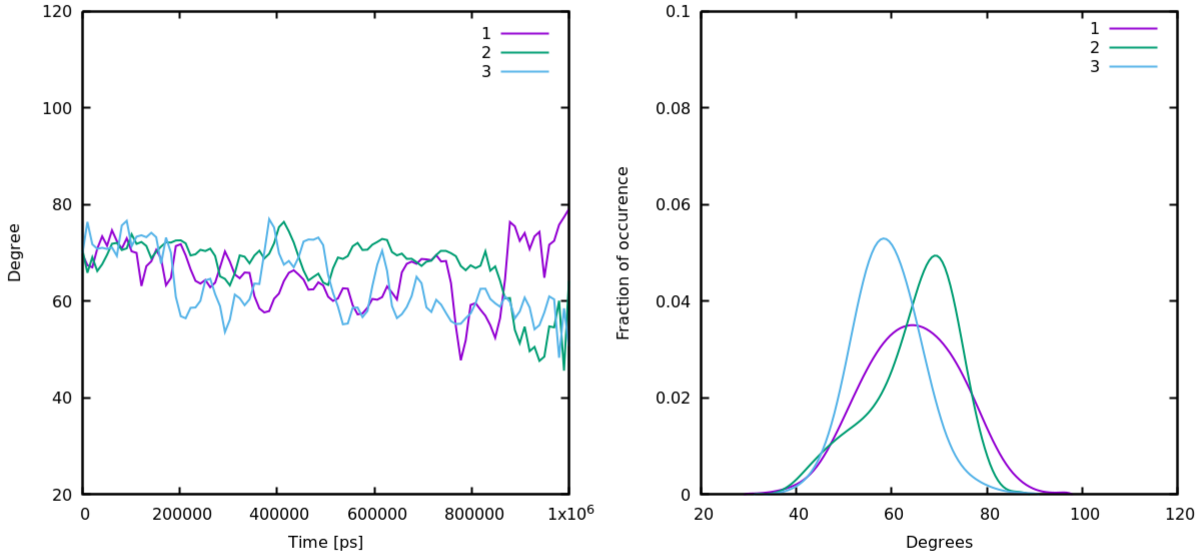

f)

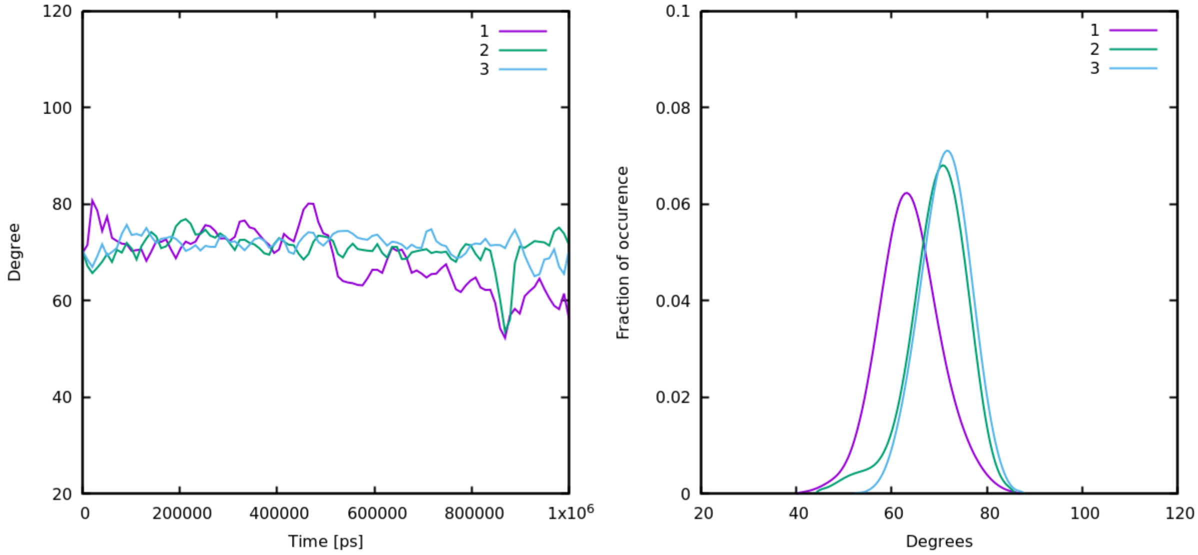

Figure S11. (continued)

Table S3. Positions of added hydrogen atoms in water molecules.

```
4HOR
HETATM 4103  O   HOH A 512       -6.647 -15.899  25.430
HETATM    0  H   HOH A 512       -7.334 -15.805  24.778
HETATM    0  H   HOH A 512       -5.783 -16.168  25.314
HETATM 4146  O   HOH A 555       -5.552 -14.449  27.798
HETATM    0  H   HOH A 555       -5.442 -13.560  28.113
HETATM    0  H   HOH A 555       -4.630 -14.912  27.698
HETATM 4503  O   HOH A 912       -7.918 -15.035  29.481
HETATM    0  H   HOH A 912       -7.245 -15.221  30.188
HETATM    0  H   HOH A 912       -8.771 -14.775  29.643

4HOS
HETATM 4033  O   HOH A 612       -6.700 -16.190  25.392
HETATM    0  H   HOH A 612       -5.838 -16.457  25.258
HETATM    0  H   HOH A 612       -7.407 -16.120  24.759
HETATM 4076  O   HOH A 655       -5.544 -14.660  27.680
HETATM    0  H   HOH A 655       -4.622 -15.121  27.567
HETATM    0  H   HOH A 655       -5.431 -13.761  27.964
HETATM 4371  O   HOH X 101       -7.854 -15.208  29.451
HETATM    0  H   HOH X 101       -7.159 -15.368  30.143
HETATM    0  H   HOH X 101       -8.704 -14.949  29.631

4HOT
HETATM 3990  O   HOH A 603      -22.155 -15.401  86.068
HETATM    0  H   HOH A 603      -21.223 -15.848  85.981
HETATM    0  H   HOH A 603      -22.063 -14.501  86.357
HETATM 4025  O   HOH A 638      -24.507 -15.988  87.770
HETATM    0  H   HOH A 638      -23.830 -16.139  88.481
HETATM    0  H   HOH A 638      -25.366 -15.741  87.925
HETATM 4026  O   HOH X 101      -23.220 -16.942  83.744
HETATM    0  H   HOH X 101      -23.909 -16.881  83.090
HETATM    0  H   HOH X 101      -22.350 -17.196  83.635
```

Table S4. Average distances [nm] between the centers of mass of chosen amino acids and nucleotides during the last 500 ns of simulations.

	IFIT5-ppp5C(C2*syn*)			IFIT5-ppp5C(C2*anti*)			IFIT5-ppp12A		
	1	2	3	1	2	3	1	2	3
Glu33	0.82 (0.05)	1.33 (0.09)	1.79 (0.08)	0.78 (0.05)	1.24 (0.10)	1.81 (0.10)	0.91 (0.06)	1.24 (0.13)	1.74 (0.12)
Gln41	1.44 (0.15)	2.04 (0.19)	2.49 (0.12)	1.43 (0.16)	1.93 (0.18)	2.51 (0.15)	1.44 (0.15)	1.86 (0.20)	2.39 (0.16)
Lys48	1.88 (0.31)	2.50 (0.34)	2.92 (0.29)	1.93 (0.36)	2.42 (0.36)	2.99 (0.32)	1.94 (0.32)	2.39 (0.35)	2.92 (0.31)
Lys150	0.78 (0.08)	1.50 (0.11)	1.95 (0.07)	0.80 (0.21)	1.40 (0.18)	2.01 (0.18)	0.83 (0.16)	1.36 (0.16)	1.92 (0.14)
Tyr185	1.13 (0.07)	1.24 (0.09)	1.42 (0.11)	1.21 (0.10)	1.21 (0.14)	1.50 (0.17)	1.04 (0.10)	1.13 (0.09)	1.38 (0.13)
Arg186	0.72 (0.06)	1.02 (0.09)	1.32 (0.10)	0.80 (0.12)	0.97 (0.14)	1.41 (0.15)	0.63 (0.12)	0.90 (0.10)	1.31 (0.11)
Asp189	1.14 (0.12)	1.00 (0.14)	1.05 (0.16)	1.17 (0.11)	0.98 (0.17)	1.12 (0.17)	1.06 (0.14)	0.95 (0.09)	1.03 (0.13)
Tyr250	1.03 (0.07)	1.43 (0.11)	1.83 (0.08)	1.10 (0.09)	1.37 (0.12)	1.87 (0.12)	1.11 (0.09)	1.34 (0.12)	1.80 (0.10)
Arg253	0.88 (0.05)	1.01 (0.11)	1.37 (0.06)	0.94 (0.07)	0.95 (0.08)	1.34 (0.11)	0.97 (0.05)	0.96 (0.08)	1.33 (0.07)
Tyr254	0.94 (0.06)	1.00 (0.09)	1.30 (0.07)	1.05 (0.09)	1.01 (0.11)	1.35 (0.14)	0.99 (0.04)	0.96 (0.07)	1.28 (0.06)
Lys257	1.22 (0.07)	0.89 (0.09)	0.97 (0.08)	1.32 (0.07)	0.97 (0.09)	0.98 (0.12)	1.23 (0.06)	0.90 (0.07)	0.93 (0.05)
Arg294	2.07 (0.07)	1.45 (0.08)	1.23 (0.12)	2.13 (0.11)	1.58 (0.10)	1.11 (0.11)	2.10 (0.07)	1.57 (0.11)	1.18 (0.08)
Asp334	1.28 (0.18)	1.36 (0.11)	1.65 (0.12)	1.27 (0.21)	1.32 (0.20)	1.60 (0.11)	1.24 (0.21)	1.20 (0.16)	1.46 (0.10)
Gln377	1.79 (0.11)	1.27 (0.13)	1.08 (0.13)	1.97 (0.16)	1.48 (0.11)	1.05 (0.14)	1.74 (0.10)	1.32 (0.12)	0.95 (0.10)

Supporting Information 2

The Excel file with calculated electrostatic interaction energies calculated with UBDB+EPMM method or point charges method for all amino acid residues for crystal structures and representative structures from six molecular dynamics simulation runs is deposited as the supplementary file "**SI_Energies**". Selected amino acid residues belonging to the binding site are marked with the number 1 in the appropriate column.
